# Inverse Problem Regularization for 3D Multi‐Species Tumor Growth Models

**DOI:** 10.1002/cnm.70057

**Published:** 2025-07-08

**Authors:** Ali Ghafouri, George Biros

**Affiliations:** ^1^ Oden Institute, University of Texas at Austin Austin Texas USA

**Keywords:** initial condition, multi‐species tumor growth model, PDE constrained optimization, regularization, single‐species model

## Abstract

We present a multi‐species partial differential equation (PDE) model for tumor growth and an algorithm for calibrating the model from magnetic resonance imaging (MRI) scans. The model is designed for glioblastoma multiforme (GBM) a fast‐growing type of brain cancer. The modeled species correspond to proliferative, infiltrative, and necrotic tumor cells. The model calibration is formulated as an inverse problem and solved by a PDE‐constrained optimization method. The data that drives the calibration is derived by a single multi‐parametric MRI image. This is a typical clinical scenario for GBMs. The unknown parameters that need to be calibrated from data include 10 scalar parameters and the infinite dimensional initial condition (IC) for proliferative tumor cells. This inverse problem is highly ill‐posed as we try to calibrate a nonlinear dynamical system from data taken at a single time. To address this ill‐posedness, we split the inversion into two stages. First, we regularize the IC reconstruction by solving a single‐species compressed sensing problem. Then, using the IC reconstruction, we invert for model parameters using a weighted regularization term. We construct the regularization term by using auxiliary 1D inverse problems. We apply our proposed scheme to clinical data. We compare our algorithm with single‐species reconstruction and unregularized reconstructions. Our scheme enables the stable estimation of non‐observable species and quantification of infiltrative tumor cells. Our regularization improves the tumor Dice score by 5%–10% compared to single‐species model reconstruction. Also, our regularization reduces model parameter reconstruction errors by 4%–80% in cases with known initial condition and brain anatomy compared to cases without regularization. Importantly, our model can estimate infiltrative tumor cells using observable tumor species.

## Graphical abstract

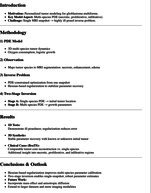

## 1 Introduction

Biophysical models of tumor growth can help quantify tumor infiltration that is not clearly visible. Quantifying infiltration is used in patient stratification, preoperative planning, and treatment planning. As every subject's anatomy and disease is unique, a main challenge is constructing subject‐specific biophysical models. This requires fitting free biophysical model parameters to data. Such parameters include the healthy brain anatomy, the location in which the tumor started, and several scalar growth coefficients modeling spreading, proliferation, and clearance. In this paper, we present an inversion methodology that integrates classical inverse problem theory with a multi‐species brain tumor growth model. Our approach addresses some challenges of single time snapshot inversion and our numerical results show strengths and weaknesses of the scheme.

### 1.1 Contributions

In our previous study [1], we developed a multi‐species tumor growth model integrated with tumor mass deformation but we did not consider its calibration from data. Calibrating this model is challenging due to the complexity of non‐linear coupled partial differential equations (PDEs) and the sparsity of the data. We are interested in reconstruction using a multiparameteric MRI scan at a single time point. We split the reconstruction into two stages. In the first stage we reconstruct the tumor initial condition (IC) assuming a simpler single species model. In the second stage, we reconstruct 10 scalar PDE coefficients using the IC from the first stage. For the first task we use our previous work on inverting for the IC using a single‐species PDE [2, 3] and use it to reconstruct model parameters. Our focus in this paper is two‐fold: (1) analyze and derive algorithms for the second stage inverse problem; and (2) evaluate the combined inversion using synthetic data. We summarize our contributions below.

1. Using a 1D model, we analyze the Hessian of the objective function (numerically), and discuss the severe ill‐posedness of the inversion problem for the 10‐parameter multi‐species PDE tumor growth model. We also use the 1D formulation to construct a weighted regularization term that we then use for 3D reconstruction. Since we do not have the ground truth parameters for clinical data, we evaluate this scheme with synthetic data. We empirically demonstrate that this regularization term improves growth coefficient estimation in the inversion setting.
2. We then estimate the tumor's IC using our previous works [2, 3]. We combine IC inversion with growth coefficients inversion. We evaluate this full scenario on synthetic data and highlight the strengths and weaknesses of the method.
3. We parallelize our execution to efficiently solve the forward problem using parallel architecture and graphics processing units (GPUs), allowing for 3D inversion on a $160^{3}$ imaging resolution to take approximately 5 h for any synthetic case on 4 GPUs.

As an example, we also report results of the reconstruction using a clinical dataset. A full clinical evaluation of the overall methodology and its comparison with a single‐species model is a subject of ongoing investigation and will be reported elsewhere.

### 1.2 Limitations

The proposed multi‐species model includes edema because it is clearly visible in multi‐parametric magnetic resonance imaging (mpMRI) scans. Our edema model is a simple algebraic relation, similar to our previously proposed model in [1]. In this analysis, we also ignore the so‐called mass effect, the normal brain tissue mechanical deformation due to the presence of the tumor. We have designed schemes to account for mechanical deformation in single‐species models ([4, 5]), but this is beyond the scope of this study. Our growth model does not incorporate diffusion tensor imaging, which can help capture more complex invasion patterns. Our single‐snapshot multiparametric MRI does not include more advanced imaging modalities like perfusion. In this paper, we use a simplified 1D model to compute the regularization, which differs from the 3D model in some terms. We estimate the tumor IC using a single‐species reaction diffusion model proposed in our previous studies [2, 3] and do not invert for the IC using the multi‐species model. Our scheme is primarily focused on brain tumors, but it is also applicable to tumors in other organs such as the pancreas, breast, prostate, and kidney.

### 1.3 Related Works

The modeling of tumor growth studies can be divided into two categories: 1. Forward models 2. Inverse problems and solution algorithms. Forward models mainly focus on the biophysical model that describes the biological process of brain tumors. Inverse problems and solution algorithms, on the other hand, involve aspects such as noise models, regularization, observation operators, and calibration of forward models in order to find the parameters to reconstruct data. Despite numerous attempts to simulate tumor growth models, less work has been done on the inverse problems themselves.

The most commonly used forward model for tumor growth is the single‐species reaction–diffusion model, as seen in various studies [6, 7, 8, 9]. Other models incorporating mass effect, chemotaxis, and angiogenesis are also explored in [10, 11, 12]. Despite offering insight into biological processes, these models lack calibration due to mathematical and computational complexities. Our group has worked to address these challenges, enabling clinical analysis of the models [5, 13].

Inverse problems have been extensively studied to calibrate single‐species reaction–diffusion models and quantify infiltrative tumor cells [14, 15, 16]. In reference [17], the inverse problem is formulated by including anisotropy diffusion derived from diffusion tensor imaging. In reference [18] the authors utilize Bayesian inversion for personalized inversion of model parameters for invasive brain tumors. Our previous works [2, 3] addressed the challenges of calibrating the growth coefficients and IC inversion simultaneously, proposing a $\ell_{0}$ constraint to localize IC inversion. In reference [4], the model is combined with mass deformation and clinical assessment is performed for a large number of MRI scans [5], improving survival prediction for patients. However, the single‐species model does not use all available information from MRI scans and considers necrotic and enhancing cells as single‐species while ignoring the edema cells, ignoring the biological process between species and resulting in a model output that differs from observed segmented tumor from MRI scans. Recent studies have incorporated data‐driven methods with follow‐up MRI validation [19, 20]. While these methods leverage larger datasets, they do not consider the coupled multi‐physics nature of tumor growth. Our approach focuses on modeling the complete multi‐physics system, though with a more limited patient cohort.

Recently [21], a dual‐species model using longitudinal MRI scans to calibrate the growth coefficients has been developed. However, longitudinal data is rarely available in clinical practice. In our previous work [1], we formulated a multi‐species model from [11] and combined it with mass deformation of the brain as a forward solver. The solver developed in this study was applied to analyze multiple clinical cases, as detailed in [22]. The present paper, however, focuses on addressing the inverse problem, with particular emphasis on examining its ill‐posed nature. Furthermore, we propose a regularization scheme to mitigate the challenges associated with ill‐posedness, an aspect not explored in our previous work [22]. This investigation aims to enhance the robustness and reliability of the solver in clinical applications.

### 1.4 Outline

In Section 2, we outline the forward problem and mathematical formulation of the inverse problem. We then present the 1D model, derive the adjoint and gradient formulations, and estimate the Hessian of the problem. We then propose an algorithm to compute a weighted regularization operator, which we then use in 3D reconstructions. The solution algorithm for the 3D inversion problem is also discussed, including the estimation of IC and growth coefficients in 3D. In Section 3, we analyze the ill‐posedness of the 1D problem and present inversion results using our computed regularization. The inversion in 3D is also analyzed for two cases: (1) estimating tumor growth parameters given IC and brain anatomy, and (2) estimating both the brain anatomy and IC while inverting for the tumor growth parameters. Results of the full inversion algorithm are presented on two clinical cases, with a comparison of the reconstructed observed species.

## 2 Methodology

In the single‐species model, we use the aggregate variable $c\left(x,t\right)$ to represent all the tumor cells. Here x is a point in ${\mathbb{R}}^{d}$ (d=3 for 3D model and d=1 for 1D model) and $t\in \left(0,T\right]$ is the time. We denote T as the time‐horizon (the time since the tumor growth onset) and we set T=1 to non‐dimensionalize PDE forward model for the single‐snapshot inversion. In the multi‐species model, we have three species: necrotic $n\left(x,t\right)$, proliferative $p\left(x,t\right)$, infiltrative $i\left(x,t\right)$. We can relate the single‐species model to the multi‐species model by setting $c\left(x,t\right)=n\left(x,t\right)+p\left(x,t\right)+i\left(x,t\right)$. We consider probability maps related to MRI scans instead of precise tissue types in our model blue where each voxel is assigned a probability value between 0 and 1 rather than a binary tissue classification. The brain is described by separate maps for gray matter ($g\left(x,t\right)$), white matter ($w\left(x,t\right)$), and cerebrospinal fluid (CSF)‐ventricles (VT), denoted by $f\left(x,t\right)$. We use diffeomorphic image registration to segment the healthy brain into gray matter, white matter, CSF and VT [13].

### 2.1 Formulation (Forward Problem)

**FIGURE 1:**
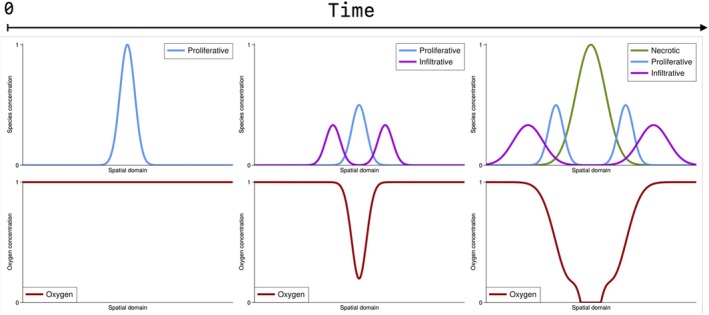
A schematic representation of the multi‐species model dynamics in 1D. The tumor species growth process is represented, with vascularization indicated by oxygen.

Our model is a go‐or‐grow multi‐species tumor model that accounts for tumor vascularization. Tumor cells are assumed to exist in one of two states: proliferative or invasive. In an environment with sufficient oxygen, tumor cells undergo rapid mitosis by consuming oxygen. However, under low oxygen concentrations (hypoxia), the cells switch from proliferative to invasive. The invasive cells migrate to richer, higher‐oxygen areas and then switch back to the proliferative state. When the oxygen levels drop below a certain level, the tumor cells become necrotic (Figure 1). Necrotic cells are dead cells that remain in this state when oxygen levels stay below the hypoxia threshold. We show the schematic progression of the multi‐species process in Figure model (presented in 1D). Initially, only proliferative cells are present, with the oxygen concentration at its maximum initial value of one. Subsequently, the proliferative cells consume the oxygen, causing them to transition into infiltrative cells. These infiltrative cells then migrate in search of additional oxygen. Our model is summarized as.

(1a) $$\partial_{t}p-ℛp+\alpha \left(o\right)p\left(1-i\right)-\beta \left(o\right)i\left(1-p\right)+\gamma \left(o\right)p\left(1-n\right)=0\;\text{in}\;\Omega \times \left(0,1\right]$$

(1b) $$\partial_{t}i-Di-ℛi-\alpha \left(o\right)p\left(1-i\right)+\beta \left(o\right)i\left(1-p\right)+\gamma \left(o\right)i\left(1-n\right)=0\;\text{in}\;\Omega \times \left(0,1\right]$$

(1c) $$\partial_{t}n-\gamma \left(o\right)\left(i+p\right)\left(1-n\right)=0\;\text{in}\;\Omega \times \left(0,1\right]$$

(1d) $$\partial_{t}o+\delta_{c}\mathrm{op}-\delta_{s}\left(1-o\right)\left(w+g\right)=0\;\text{in}\;\Omega \times \left(0,1\right]$$

(1e) $$\partial_{t}w+\frac{w}{w+g}\left(Di+ℛp+ℛi\right)=0\;\text{in}\;\Omega \times \left(0,1\right]$$

(1f) $$\partial_{t}g+\frac{g}{w+g}\left(Di+ℛp+ℛi\right)=0\;\text{in}\;\Omega \times \left(0,1\right]$$

(1g) $$p\left(x,0\right)-p_{0}=0\;\text{in}\;\Omega$$

(1h) $$i\left(x,0\right)=0\;\text{in}\;\Omega$$

(1i) $$n\left(x,0\right)=0\;\text{in}\;\Omega$$

(1j) $$o\left(x,0\right)-1=0\;\text{in}\;\Omega$$

(1k) $$g\left(x,0\right)-g_{0}=0\;\text{in}\;\Omega$$

(1l) $$w\left(x,0\right)-w_{0}=0\;\text{in}\;\Omega$$

(1m) $$f\left(x,0\right)-f_{0}=0\;\text{in}\;\Omega$$

The paper uses notations listed in Table 1. The model ignores mass effects from proliferative and necrotic cells and uses conservation equations with transitional (γ,β and α), source ($\delta_{c}$, $\delta_{s}$, and ℛ), and diffusion (D) terms. See [1, 11] for model choice and description. The reaction operator (ℛ) for species s is modeled as an inhomogeneous nonlinear logistic growth operator as,
(2)

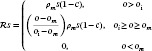

where $o_{m}$ and $o_{i}$ are the mitosis and invasive oxygen thresholds, respectively. We restrict the growth conditions with different oxygen levels. We use $o_{m}=\frac{o_{i}+o_{h}}{2}$ from [1, 11]. $\rho_{m}$ is an inhomogeneous spatial growth term defined as,

(3) $$\rho_{m}\left(x\right)=\mathrm{\rho w}+\rho_{g}g,$$

where ρ and $\rho_{g}$ are scalar coefficient quantifying proliferation in white matter and gray matter, respectively. We assume 20% growth in gray matter ($\rho_{g}=0.2\rho$) compared to white matter [18, 23, 24]. The growth terms converge to zero when the total tumor concentration (c) reaches 1. The inhomogeneous isotropic diffusion operator D is defined as follows.

(4) $$Di=\mathrm{div}\left(k\nabla i\right)$$

where k defines the inhomogeneous diffusion rate in gray and white matter and we define it as,

(5) $$k=\mathrm{\kappa w}+\kappa_{g}g$$

where κ and $\kappa_{g}$ are scalar coefficient quantifying diffusion in white matter and gray matter, respectively. We set $\kappa_{g}=0.2\kappa$ [18, 23, 24]. Note that the migration and proliferation occur only into white/gray matter and not in CSF/VT. The parameters α,β, and γ functions between are set as follows,

(6) $$\alpha \left(o\right)=\alpha_{0}ℋ\left(o_{i}-o\right)$$

(7) $$\beta \left(o\right)=\beta_{0}ℋ\left(o-o_{i}\right)$$

(8) $$\gamma \left(o\right)=\gamma_{0}ℋ\left(o_{h}-o\right)$$

where the scalar coefficients controlling species transitioning are $\alpha_{0},\beta_{0}$, and $\gamma_{0}$, and ℋ is the smoothed Heaviside function. There are no movement equation for ventricles since our model does not include mass deformation due to tumor growth. Note that our model differs from the one in [1] in the following ways:

- The transition term αp is changed to $\mathrm{\alpha p}\left(1-i\right)$ to prevent transitioning once i≈1 (See Equations (1a) and (1b)).
- The transition term βi is changed to $\mathrm{\beta i}\left(1-p\right)$ to prevent transitioning once p≈1 (See Equations (1a) and (1b)).
- The necrosis term γi and γp are changed to $\mathrm{\gamma i}\left(1-n\right)$ and $\mathrm{\gamma p}\left(1-n\right)$ to prevent death once n≈1 (See Equations ((1a), (1b), (1c))).
- A term in reaction operator ℛ on a species s is changed from $\left(1-s\right)$ to $\left(1-c\right)$, where c is the total tumor concentration $\left(c=i+p+n\right)$. This change ensures that there will be no reaction or increase of tumor concentration once the total tumor concentration c≈1 (See Equations (1a), (1b), (1e), and (1f)).
- In Equation (1c), the term $\gamma \left(g+w\right)$ has been removed to ensure that only infiltrative and proliferative cells can transition into the necrotic state.
- We modify the β function to be the complement of the α function (See Equation (7)) in oxygen terms.
- We have incorporated the edema model into the observation operator instead of using a separate model (See Equation (12)).

**TABLE 1:** Common notations used to describe the forward and inverse problem for the multi‐species tumor growth model.

Notation	Description	Range
x	Spatial coordinates	(${\left[0,2\pi \right]}^{3}$ for 3D and $\left[0,2\pi \right]$ in 1D)
t	Time	$\left[0,1\right]$
p	Proliferative tumor cells	$\left[0,1\right]$
i	Infiltrative tumor cells	$\left[0,1\right]$
n	Necrotic tumor cells	$\left[0,1\right]$
c	Tumor cell (c=i+p+n)	$\left[0,1\right]$
o	Oxygen concentration	$\left[0,1\right]$
g	Gray matter cells	$\left[0,1\right]$
w	White matter cells	$\left[0,1\right]$
f	CSF/VT	$\left[0,1\right]$
$p_{0}$	Initial condition (IC) for proliferative cells ($p\left(x,0\right)$) (See Equation (1g))	$\left[0,1\right]$
ℛ	Tumor growth operator (logistic growth operator)	—
D	Tumor migration operator (diffusion)	—
$\rho_{m}$	Inhomogeneous reaction rate	—
k	Inhomogeneous diffusion rate	—
$\alpha \left(o\right)$	Transition function from p to i cells	—
$\beta \left(o\right)$	Transition function from i to p cells	—
$\gamma \left(o\right)$	Transition function from i and p to n cells	—
ρ	Reaction coefficient (See Equation (2))	$\left[5.0,,,2.50,\times ,10^{1}\right]$
κ	Diffusion coefficient (See Equation (4))	$\left[1,\times ,10^{-3},,,1,\times ,10^{-1}\right]$
$\alpha_{0}$	Transitioning coefficient from p to i cells (See Equation (6))	$\left[1,\times ,10^{-1},,,1.00,\times ,10^{1}\right]$
$\beta_{0}$	Transitioning coefficient from i to p cells (See Equation (7))	$\left[1,\times ,10^{-1},,,1.50,\times ,10^{1}\right]$
$\gamma_{0}$	Transitioning coefficient from i and p to n cells (See Equation (8))	$\left[1.0,,,2.00,\times ,10^{1}\right]$
$\delta_{c}$	Oxygen consumption rate (See Equation (1d))	$\left[1.0,,,2.00,\times ,10^{1}\right]$
$\delta_{s}$	Oxygen supply rate (See Equation (1d))	$\left[1.0, 8.0\right]$
$o_{h}$	Hypoxic oxygen threshold (See Equations (2) and (8))	$\left[1,\times ,10^{-3},,,8,\times ,10^{-1}\right]$
$o_{i}$	Invasive oxygen threshold (See Equations (2), (6), and (7))	$\left[2,\times ,10^{-1},,,1.0\right]$
$i_{e}$	Threshold coefficient for edema (See Equation (12))	$\left[1,\times ,10^{-3},,,3,\times ,10^{-1}\right]$

### 2.2 Observation Operators

We now discuss how we relate problem predictions to MRI images. Currently, MRI cannot fully reveal tumor infiltration. Instead, we use a relatively standard preprocessing workflow [25] in which the MRI is segmented to different tissue types. For each patient, the preprocessing workflow includes affine registration of the four mpMRI scans to a template atlas, followed by tumor region segmentation using a neural network trained on the BraTS dataset with expert radiologist labels. We define the tumor region and segment healthy substructures (GM, WM, VT, CSF) using ANTs [26] with ensemble atlas‐based segmentation, with these segmentation labels serving as proxies for tissue concentration data. To reconcile the species concentrations generated by our multi‐species model with the observed species segmentation obtained from MRI scans, we need to introduce additional modeling assumptions related to the so‐called *observation operator*. These operators map the model‐predicted fields to a binary (per tissue type) segmentation, which can then be compared to the MRI segmentation. In particular, we define observation operators for the total tumor region (c) and each individual species, allowing us to determine the most probable species at each point x. The observation operators are defined as follows:

(9) $$Oc=ℋ\left(c-w\right)ℋ\left(c-g\right)ℋ\left(c-f\right)$$

(10) $$Op=ℋ\left(p-n\right)ℋ\left(p-i\right)Oc$$

(11) $$On=ℋ\left(n-p\right)ℋ\left(n-i\right)Oc$$

(12) $$Ol=\left(1-Op-On\right)ℋ\left(i-i_{e}\right)$$

where Oc,Op,On, and Ol are the observed tumor (c), proliferative (p), necrotic (n) and edema cells (l), respectively. Note that the species used in the above setup are the species at t=1. In this setup, we first determine the most probable location for the tumor (c) using Equation (9), and then we determine the most probable species within Oc using Equations (10) and (11). To model edema, we adopt a simplified approach similar to [1]. In this model, locations with infiltrative concentration above a threshold ($i_{e}$) are considered as edema if they are not detected as necrotic or proliferative. This model treats edema as a label that is independent of the other PDEs, and can thus be computed in the observation operator Ol using Equation (12). To model the Heaviside function ℋ, we use a sigmoid approximation function given by,

(13) $$ℋ\left(x\right)=\frac{1}{1+e^{-\mathrm{\omega x}}}$$

where ω is the shape factor, determining the smoothness of the approximation. This parameter is set based on the spatial discretization. Thus, the proposed observation operators do not have any free parameters. Next, we discuss the inverse problem to invert for the model parameters.

### 2.3 Inverse Problem

Our model requires three parameters to characterize tumor progression:

- Healthy precancerous brain segmentation (Ω).
- IC of proliferative tumor cells ($p_{0}$).
- Growth coefficients vector denoted by q consisting of the following terms: diffusion coefficient κ in Equation (4), reaction coefficient ρ in Equation (2), transition coefficients $\alpha_{0}$, $\beta_{0}$, and $\gamma_{0}$ in Equations ((6), (7), (8)), oxygen consumption rate $\delta_{c}$ in Equation (1d), oxygen supply rate $\delta_{s}$ in Equation (1d), hypoxic oxygen threshold $o_{h}$ in Equation (2) and (8), invasive oxygen threshold in Equations (2), (6), and (7) and threshold coefficient for edema $i_{e}$ in Equation (12).

We discuss in Sections 2.5.1 and 2.5.2 how we estimate Ω and $p_{0}$. Here, we focus on growth coefficients q inversion. Given the Ω and $p_{0}$, we frame the inverse problem as a constrained optimization problem,
(14)

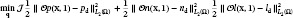

(15) $$s.t.\;ℱ\left(p_{0},q\right)\;\text{in}\;\Omega \times \left(0,1\right]$$

We aim to minimize the objective function J by optimizing the scalar coefficients vector q in the forward model $ℱ\left(p_{0},q\right)$. This inverse problem is a generalization of the single‐species reaction–diffusion PDE model that has already been shown to be exponentially ill‐posed [2, 27, 28, 29]. We circumvent the ill‐posedness by designing a problem regularization that involves a combination of sparsity constraints, a max constraint in the initial condition and $\ell_{2}$ penalty on the unknown parameters, κ and ρ. As we have more undetermined parameters, we cannot use the regularization scheme in [2, 3]. To address this ill‐posedness, we first perform analysis on the Hessian of the optimization problem and then generalize it to the full 3D inversion model. Next, we perform an analysis for the 1D inverse problem and compute a regularization term to assist in estimation of growth coefficients q.

### 2.4 Coefficients Inversion Analysis for a 1D Model

To demonstrate the ill‐posedness of our model, we analyze a multi‐species model in 1D. To simplify the problem, we assume that the only normal tissue type is white matter (removing the gray matter, ventricles (VT), and CSF from the model). In this setting, the forward model with periodic boundary conditions is described by:

(16a) $$\partial_{t}p-ℛp+\mathrm{\alpha p}\left(1-i\right)-\mathrm{\beta i}\left(1-p\right)+\mathrm{\gamma p}\left(1-n\right)=0\;\text{in}\;\Omega \times \left(0,1\right]$$

(16b) $$\partial_{t}i-Di-ℛi-\mathrm{\alpha p}\left(1-i\right)+\mathrm{\beta i}\left(1-p\right)+\mathrm{\gamma i}\left(1-n\right)=0\;\text{in}\;\Omega \times \left(0,1\right]$$

(16c) $$\partial_{t}n-\gamma \left(i+p\right)\left(1-n\right)=0\;\text{in}\;\Omega \times \left(0,1\right]$$

(16d) $$\partial_{t}o+\delta_{c}\mathrm{op}-\delta_{s}\left(1-o\right)w=0\;\text{in}\;\Omega \times \left(0,1\right]$$

(16e) $$\partial_{t}w+Di+ℛp+ℛi=0\;\text{in}\;\Omega \times \left(0,1\right]$$

(16f) $$p\left(x,0\right)-p_{0}=0\;\text{in}\;\Omega$$

(16g) $$i\left(x,0\right)=0\;\text{in}\;\Omega$$

(16h) $$n\left(x,0\right)=0\;\text{in}\;\Omega$$

(16i) $$o\left(x,0\right)=1\;\text{in}\;\Omega$$

(16j) $$w\left(x,0\right)-1+p_{0}=0\;\text{in}\;\Omega$$

The optimization problem has exactly the same form as Equations (14) and (15),
(17)

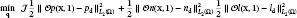

(18) $$s.t.ℱ\left(p_{0},q\right)\;\text{in}\;\Omega \times \left(0,1\right]$$

where $p_{d},n_{d}$, and $l_{d}$ are the proliferative, necrotic, and edema data. The corresponding Lagrangian of this problem is given by:
(19)

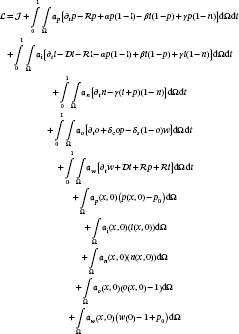

where $a_{p},a_{i},a_{n},a_{o}$, and $a_{w}$ are the adjoint variables with respect to p,i,n,o, and w.

The first order optimality conditions can be derived by requiring stationary of the Lagrangian with respect to the state variables (p,i,n,o, and w). Therefore, we obtain,

(20a) $$\frac{\mathrm{\partial ℒ}}{\partial p}=0$$

(20b) $$\frac{\mathrm{\partial ℒ}}{\partial i}=0$$

(20c) $$\frac{\mathrm{\partial ℒ}}{\partial n}=0$$

(20d) $$\frac{\mathrm{\partial ℒ}}{\partial o}=0$$

(20e) $$\frac{\mathrm{\partial ℒ}}{\partial w}=0$$

Therefore, the adjoint equations are

(21a) $$\begin{array}{r} -\partial_{t}a_{p}-\frac{\mathrm{\partial ℛ}p}{\partial p}\left(a_{p}-a_{w}\right)+\alpha \left(1-i\right)\left(a_{p}-a_{i}\right)+\mathrm{\beta i}\left(a_{p}-a_{i}\right)+\gamma \left(1-n\right)\left(a_{p}-a_{n}\right)\\ -\frac{\mathrm{\partial ℛ}i}{\partial p}\left(a_{i}-a_{w}\right)+\delta_{c}oa_{o}=0\;\text{in}\;\Omega \times \left(0,1\right] \end{array}$$

(21b) $$a_{p}\left(x,1\right)+\left(Op-p_{d}\right)\frac{\partial Op}{\partial p}+\left(On-n_{d}\right)\frac{\partial On}{\partial p}+\left(Ol-l_{d}\right)\frac{\partial Ol}{\partial p}=0\;\text{in}\;\Omega$$

(21c) $$\begin{array}{l} -\partial_{t}a_{i}-{Da}_{i}+{Da}_{w}+\mathrm{\alpha p}\left(a_{i}-a_{p}\right)+\beta \left(1-p\right)\left(a_{i}-a_{p}\right)+\gamma \left(1-n\right)\left(a_{i}-a_{n}\right)\\ -\frac{\mathrm{\partial ℛ}i}{\partial i}\left(a_{i}-a_{w}\right)-\frac{\mathrm{\partial ℛ}p}{\partial i}\left(a_{p}-a_{w}\right)=0\;\text{in}\;\Omega \times \left(0,1\right] \end{array}$$

(21d) $$a_{i}\left(x,1\right)+\left(Op-p_{d}\right)\frac{\partial Op}{\partial i}+\left(On-n_{d}\right)\frac{\partial On}{\partial i}+\left(Ol-l_{d}\right)\frac{\partial Ol}{\partial i}=0\;\text{in}\;\Omega$$

(21e) $$\begin{array}{l} -\partial_{t}a_{n}+\gamma \left(\left(i+p\right)a_{n}-ia_{i}-pa_{p}\right)-\frac{\mathrm{\partial ℛ}p}{\partial n}\left(a_{p}-a_{w}\right)\\ -\frac{\mathrm{\partial ℛ}i}{\partial n}\left(a_{i}-a_{w}\right)=0\;\text{in}\;\Omega \times \left(0,1\right] \end{array}$$

(21f) $$a_{n}\left(x,1\right)+\left(Op-p_{d}\right)\frac{\partial Op}{\partial n}+\left(On-n_{d}\right)\frac{\partial On}{\partial n}+\left(Ol-l_{d}\right)\frac{\partial Ol}{\partial n}=0\;\text{in}\;\Omega$$

(21g) $$\begin{array}{l} -\partial_{t}a_{o}+\frac{\partial \gamma }{\partial o}\left(1-n\right)\left(ia_{i}+pa_{p}-\left(i+p\right)a_{n}\right)+\frac{\partial \alpha }{\partial o}p\left(1-i\right)\left(a_{p}-a_{i}\right)-\frac{\partial \beta }{\partial o}i\left(1-p\right)\left(a_{p}-a_{i}\right)\\ +\delta_{c}pa_{o}+\delta_{s}wa_{o}-\frac{\mathrm{\partial ℛ}p}{\partial o}\left(a_{p}-a_{w}\right)-\frac{\mathrm{\partial ℛ}i}{\partial o}\left(a_{i}-a_{w}\right)=0\;\text{in}\;\Omega \times \left(0,1\right] \end{array}$$

(21h) $$a_{o}\left(x,1\right)=0\;\text{in}\;\Omega$$

(21i) $$\begin{array}{l} -\partial_{t}a_{w}+\frac{\partial Di}{\partial w}\left(a_{w}-a_{i}\right)-\delta_{c}\left(1-o\right)a_{o}+\frac{\mathrm{\partial ℛ}p}{\partial w}\left(a_{w}-a_{p}\right)\\ +\frac{\mathrm{\partial ℛ}i}{\partial w}\left(a_{w}-a_{i}\right)=0\;\text{in}\;\Omega \times \left(0,1\right] \end{array}$$

(21j) $$a_{w}\left(x,1\right)+\left(Op-p_{d}\right)\frac{\partial Op}{\partial w}+\left(On-n_{d}\right)\frac{\partial On}{\partial w}+\left(Ol-l_{d}\right)\frac{\partial Ol}{\partial w}=0\;\text{in}\;\Omega$$

And finally, the inversion (gradient) equations for the parameters can be evaluated as,

(22a) $$\frac{\mathrm{\partial ℒ}}{\partial \kappa }=\int_{0}^{1}\int_{\Omega }-\frac{\partial Di}{\partial \kappa }\left(a_{i}-a_{w}\right)\;\mathrm{d\Omega}\;dt$$

(22b) $$\frac{\mathrm{\partial ℒ}}{\partial \rho }=\int_{0}^{1}\int_{\Omega }-\frac{\mathrm{\partial ℛ}p}{\partial \rho }\left(a_{p}-a_{w}\right)-\frac{\mathrm{\partial ℛ}i}{\partial \rho }\left(a_{p}-a_{w}\right)\;\mathrm{d\Omega}\;dt$$

(22c) $$\frac{\mathrm{\partial ℒ}}{\partial \beta_{0}}=\int_{0}^{1}\int_{\Omega }\frac{\partial \beta }{\partial \beta_{0}}i\left(1-p\right)\left(a_{i}-a_{p}\right)\;\mathrm{d\Omega}\;dt$$

(22d) $$\frac{\mathrm{\partial ℒ}}{\partial \alpha_{0}}=\int_{0}^{1}\int_{\Omega }\frac{\partial \alpha }{\partial \alpha_{0}}p\left(1-i\right)\left(a_{p}-a_{i}\right)\;\mathrm{d\Omega}\;dt$$

(22e) $$\frac{\mathrm{\partial ℒ}}{\partial \gamma_{0}}=\int_{0}^{1}\int_{\Omega }\frac{\partial \gamma }{\gamma_{0}}\left(1-n\right)\left(pa_{p}-pa_{n}+ia_{i}-ia_{n}\right)\;\mathrm{d\Omega}\;dt$$

(22f) $$\frac{\mathrm{\partial ℒ}}{\partial \delta_{c}}=\int_{0}^{1}\int_{\Omega }a_{o}\mathrm{op}\;\mathrm{d\Omega}\;dt$$

(22g) $$\frac{\mathrm{\partial ℒ}}{\partial \delta_{s}}=\int_{0}^{1}\int_{\Omega }-a_{o}\left(1-o\right)w\;\mathrm{d\Omega}\;dt$$

(22h) $$\frac{\mathrm{\partial ℒ}}{\partial o_{i}}=\int_{0}^{1}\int_{\Omega }\frac{\partial \gamma }{\partial o_{h}}\left(1-n\right)\left(pa_{p}-pa_{n}+ia_{i}-ia_{n}\right)\;\mathrm{d\Omega}\;dt$$

(22i) $$\frac{\mathrm{\partial ℒ}}{\partial o_{h}}=\int_{0}^{1}\int_{\Omega }\frac{\partial \gamma }{\partial o_{h}}\left(1-n\right)\left(pa_{p}-pa_{n}+ia_{i}-ia_{n}\right)\;\mathrm{d\Omega}\;dt$$

(22j) $$\frac{\mathrm{\partial ℒ}}{\partial i_{e}}=\int_{\Omega }-\left(Ol\left(x,1\right)-l_{d}\right)\left(1-Op-On\right){ℋ}^{\prime }\left(i-i_{e}\right)\;dx$$

where ${ℋ}^{\prime }$ is the derivative of the Heaviside function.

To compute the gradient, it is necessary to first solve the forward problem, as outlined in Equations ((16a), (16b), (16c), (16d), (16e), (16f), (16g), (16h), (16i), (16j)), then solve the adjoint equations, as per Equations ((21a), (21b), (21c), (21d), (21e), (21f), (21g), (21h), (21i), (21j)), and then compute the gradient using Equations ((22a), (22b), (22c), (22d), (22e), (22f), (22g), (22h), (22i), (22j)). To empirically study the ill‐posedness of the 1D inversion problem, we compute the Hessian of the optimization setup through central differencing of the gradients computed in Equations ((22a), (22b), (22c), (22d), (22e), (22f), (22g), (22h), (22i), (22j)).

**FIGURE 2:**
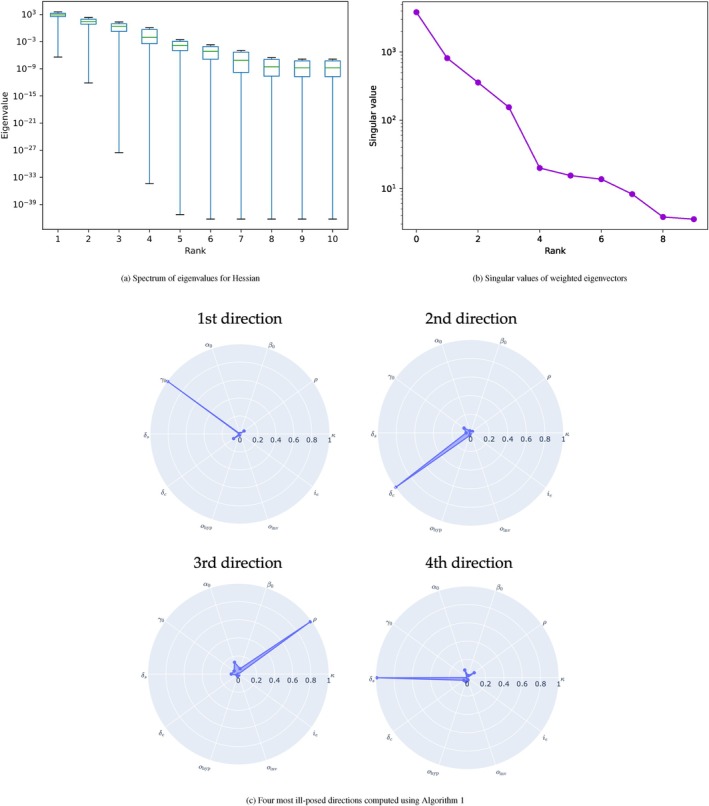
Ill‐posedness analysis of multi‐species model in 1D. The spectrum of Hessian eigenvalues for 3500 parameter combinations is presented in Figure 2a. The singular values calculated in Algorithm 1 are shown in Figure 2b. We depict the absolute value of last four directions of the computed regularization term ($U_{R}$) in Figure 2c corresponding to the four smallest singular values. The exponential decrease of eigenvalues in Figure 2a indicates high ill‐posedness of the problem. Additionally, the wide range of small eigenvalues suggests varying degrees of ill‐posedness among samples, which is accounted for in Algorithm 1. Figure 2b demonstrates how different directions impact the ill‐posedness, with smaller singular values indicating greater ill‐posedness. Thus, using the inverse of the singular directions can lead to improved inversion results. The results in Figure 2c clearly indicates ill‐posedness for high values $\gamma_{0}$ and a combination of high values of ρ and high values of $\delta_{c}$ contributes to ill‐posedness.

In Section 3.3.1, the results of the ill‐posedness analysis are presented and the spectrum of eigenvalues of the Hessian for samples (q) from coefficient space is illustrated in Figure 2a. To overcome this challenge, a regularization method is proposed in Algorithm 1 to penalize these ill‐posed directions in the objective function. The method begins by uniformly sampling from the growth coefficient space and estimating the Hessian at the minimum objective function value for each sample. The samples with smaller eigenvalues are deemed to contain less valuable information and, thus, their corresponding eigenvectors are weighted accordingly. A singular value decomposition is then performed to evaluate the weight of ill‐posed directions among different coefficient combinations. The singular values are depicted in Figure 2b. The right singular directions are identified and weighted based on the inverse of the singular values ($U_{R}$) to mitigate directions with small singular values in the objective function. These directions with smaller singular values are more ill‐posed and, therefore, require more penalization in the objective function.

Specifically, we uniformly sample N=3500 parameter values and compute the Hessian matrix $H\left(q\right)$ at each objective function minimum. We then perform singular value decomposition on these Hessian matrices ($H=U\Sigma V^{T}$) to identify the principal directions of ill‐posedness. From this decomposition, we construct the regularization operator ${U_{R}}^{T}q$, which we incorporate into the regularized objective function:

(23) $$J_{\mathrm{reg}}\left(q\right)=J\left(q\right)+\frac{\lambda }{2}∥{U_{R}}^{T}q{∥}^{2}$$

where λ controls the regularization strength. The fast decay of the Hessian eigenvalues shown in Figure 2a indicates high ill‐posedness of the problem. Additionally, the wide range of small eigenvalues suggests varying degrees of ill‐posedness among samples, which is accounted for in our regularization approach. Figure 2b demonstrates how different directions impact the ill‐posedness, with smaller singular values indicating greater ill‐posedness.

ALGORITHM 1 **Algorithm to compute the regularization.**

1: **procedure** (n, $b_{l},$ $b_{u}$)         ⊳ Get the number of samples n, lower bound vector $b_{l}$ and upper bounds vector $b_{u}$

2:     ${\left\{q_{i}\right\}}_{i=1}^{n}∼U\left(b_{l},b_{u}\right)$                          ⊳ Take uniform samples of parameters

3:     D←0

4:     **for** i=1…n **do**

5:         $H\leftarrow \text{Hess}\left(q_{i}\right)$                                    ⊳ Compute the Hessian

6:        $\Lambda ,Q\leftarrow \text{EvalDecomp}\left(H\right)$                    ⊳ Compute Evals. and Evecs. of Hessian

7:        $D\leftarrow D\cup \Lambda^{\frac{1}{2}}Q$                         ⊳ Store the weighted ill‐posed directions

8:     **end for**

9:     $\Sigma ,U\leftarrow \mathrm{SVD}\left(D\right)$                                 ⊳ SVD on ill‐posed directions

10:    $U_{R}\leftarrow U\Sigma^{-1}$                                ⊳ Weighting the ill‐posed directions

11: **end procedure**

### 2.5 3D Inversion

As mentioned, the inverse problem requires three parameters to characterize the tumor progression: (a) Healthy brain anatomy Ω to describe $w\left(x,0\right),g\left(x,0\right),f\left(x,0\right)$. (b) Initial condition of proliferative tumor cells $p_{0}$ and (c) growth coefficients vector q. In the following, we explain how we estimate each of these parameters.

#### 2.5.1 Brain Anatomy

The process of deriving the healthy brain anatomy (Ω) involves multiple steps. First, an MRI scan is affine registered to a template atlas. Then, a neural network [18] trained on the BraTS dataset [25] is used to segment the tumor into proliferative, necrotic, and edema regions. Next, 10 normal brain scans with known healthy segmentation are registered to the patient's scan using [26]. An ensemble‐based approach is then used to combine the atlas‐based segmentations for the different healthy tissue labels [5, 13]. We combine the healthy tissue labels and tumor segmentation as a single brain segmentation of the patient. Finally, the tumorous regions are replaced with white matter to estimate the healthy brain anatomy Ω.

#### 2.5.2 IC Inversion

To estimate IC of proliferative tumor cells, we use the single‐species IC inversion proposed in [3]. This algorithm uses an adjoint based method for reaction–diffusion PDE model constrained with sparsity and max constraints. Our current inverse IC scheme deviates from the [3] algorithm by limiting the support region to the necrotic areas only, as our simulations in [1] revealed that the IC always present in the necrotic regions.

#### 2.5.3 Coefficients Inversion

To invert for the growth coefficients q in 3D, we use the regularization term computed from the 1D model as sampling the Hessian is very expensive in 3D. As the structure of the nonlinear operators is the same, our hypothesis is the 1D regularization operator can be used to stabilize the 3D reconstruction. One contribution of this paper is to verify the hypothesis using numerical experiments with synthetically constructed data. Given the IC ($p_{0}$) and brain anatomy (Ω) estimated from Sections 2.5.1 and 2.5.2, we can solve the following optimization problem with constraints:

(24) $$\begin{array}{cc} \min_{q}J= & \;\frac{1}{2}∥Op\left(x,1\right)-p_{d}{∥}_{L_{2}\left(\Omega \right)}^{2}+\frac{1}{2}∥On\left(x,1\right)-n_{d}{∥}_{L_{2}\left(\Omega \right)}^{2}\\ & \;+\frac{1}{2}∥Ol\left(x,1\right)-l_{d}{∥}_{L_{2}\left(\Omega \right)}^{2}+\frac{\lambda }{2}∥{U_{R}}^{T}q{∥}^{2} \end{array}$$

(25) $$\text{subject to}\;ℱ\left(p_{0},q\right)\;\text{in}\;\Omega \times \left(0,1\right]$$

## 3 Results

In this study, we evaluate our algorithm on synthetic cases and clinical dataset. We carry out the 3D inversions using the Frontera and Lonestar system at the Texas Advanced Computing Center (TACC) at The University of Texas at Austin. Our solver is written in both C++ and Python. The forward solver uses C++ with PETSc, AccFFT, PnetCDF. The inverse problem is solved using the covariance matrix adaptation evolution strategy (CMA‐ES) in Python [30, 31]. Each 3D inversion takes 32 h on a single NVIDIA Quadro RTX 5000 GPU. For 1D test‐cases, we use Python for the forward problem with the same CMA‐ES optimizer.

### 3.1 Numerical Parameters

We list all numerical parameters used in our solver in Table 2. We now explain a justification for our choice:

- For the multi‐species forward solver, we use the same setting and numerics of [3].
- For IC inversion, we use the solver from our previous studies [2, 3]. The single‐species reaction diffusion model with $\ell_{0}$ constraint is used to solve a single‐snapshot inverse problem iteratively. The IC is estimated within necrotic regions and the sparsity parameter is fixed at 5 to allow up to 5 voxels to be considered for IC. The experiments on choosing this value can be found in [3].
- We choose $160^{3}$ as spatial discretization for 3D inversion to balance computational cost with detail preservation, reducing computation from standard $256^{3}$ while maintaining tumor characteristics and volume measurements.
- We determine the optimal value for the regularization parameter λ by evaluating the quality of data reconstruction with 20% additive noise. We select this noise percentage because previous studies have demonstrated that the tumor segmentation tool [25] achieves an accuracy of approximately 80%.
- For the inversion of growth coefficients q, we employ CMA‐ES with a relative function tolerance of $1\times 10^{-8}$ and an absolute function tolerance of $1\times 10^{-3}$. Our analysis indicates that reducing these tolerance values further does not result in improved outcomes.
- The sample size per iteration for the CMA‐ES is 16 as a balance between evaluation costs and having a diverse population for covariance sampling.
- We set the initial guess for the CMA‐ES solver to the midpoint of the parameter bounds and the initial variance to half the parameter range. Despite experimenting with higher variances and different initial guesses, we did not observe any improvement in solver performance.
- We use a shape factor ω of 32 for all the Heaviside functions except for $ℋ\left(i-i_{e}\right)$ to achieve a smooth transition. For the edema region, a smooth Heaviside function in $ℋ\left(i-i_{e}\right)$ can lead to high spatial diffusivity of infiltrative cells i. To prevent this, we use a larger shape factor (ω=256) for the edema region compared to the others. We did not observe sensitivity to this parameter in our results.

**TABLE 2:** Numerical parameters for tumor inversion and forward solver. Initial guess is mid‐range for growth coefficients. We refer to [3] for IC inversion parameters in more details.

Parameter	Value
Spatial discretization for 1D inversion	256
Spatial discretization for 3D inversion	$160^{3}$
Time‐step of the forward solvers in 1D and 3D	$2\times 10^{-2}$
Shape factor ω in Oc, On,Op,α,β and γ	32
Shape factor ω in $ℋ\left(i-i_{e}\right)$	256
Epsilon used in finite differencing of Hessian, ϵ	$1\times 10^{-6}$
Regularization parameter in 1D inversion, λ	$1\times 10^{-4}$
Regularization parameter in 3D inversion, λ	$1\times 10^{-5}$
Relative function tolerance	$1\times 10^{-7}$
Absolute function tolerance	$1\times 10^{-3}$
Sample size per iteration for the CMA‐ES solver	16

### 3.2 Performance Measures

Our main goal of the numerical experiments is to show how the proposed regularization can stabilize the multi‐species inversion and then use the same regularization to perform a full clinical inversion. First, we show how the regularization helps the inversion in synthetic cases and then combine it with IC inversion on clinical scans. In this regard, we define the following metrics to quantify our reconstructions,

- relative error in growth coefficient ι:
(26) $$e_{\iota }=\frac{|\iota^{\mathrm{rec}}-\iota^{*}|}{|\iota^{*}|}$$
  where $\iota^{*}$ is ground truth coefficient and $\iota^{\mathrm{rec}}$ is the reconstructed value. We denote the growth coefficients vector with q and $e_{q}$ denotes the average relative error of growth coefficients.
- relative error in final species reconstruction $s\left(t=1\right)$ where s can be n, p or i:
(27) $$\mu_{s,L_{2}}=\frac{∥s^{\mathrm{rec}}\left(1\right)-s^{*}\left(1\right){∥}_{L_{2}\left(\Omega \right)}}{∥s^{*}\left(1\right){∥}_{L_{2}\left(\Omega \right)}}$$
  where $s^{\mathrm{rec}}\left(1\right)$ is the final reconstructed species, $s^{*}\left(1\right)$ is the final species concentration grown with ground truth growth coefficients $q^{*}$.
- relative error in initial tumor concentration:
(28) $$\mu_{0,L_{2}}=\frac{∥p_{0}^{\mathrm{rec}}-p_{0}^{*}{∥}_{L_{2}\left(\Omega \right)}}{∥p_{0}^{*}{∥}_{L_{2}\left(\Omega \right)}}$$
  where $p^{\mathrm{rec}}\left(0\right)$ is the reconstructed IC and $p^{*}\left(0\right)$ is the ground truth IC.
- relative $\ell_{1}$ error in initial tumor parameterization ($p_{0}$):
(29) $$\mu_{0,\ell_{1}}=\frac{∥p_{0}^{\mathrm{rec}}-p_{0}^{*}{∥}_{\ell_{1}}}{∥p_{0}^{*}{∥}_{\ell_{1}}}$$
  where $p_{0}^{\mathrm{rec}}$ is the reconstructed tumor IC and $p_{0}^{*}$ is the ground truth IC.
- relative $\ell_{1}$ reconstruction for projected IC along a axis:
(30) $$\mu_{0,\ell_{1}}^{a}=\frac{∥\int_{a}\left(p_{0}^{\mathrm{rec}}-p_{0}^{*}\right)da{∥}_{\ell_{1}}}{∥\int_{a}p_{0}^{*}da{∥}_{\ell_{1}}}$$
  where a represents the axis which can be x,y or z.
- reconstruction Dice score for an observed species s:
(31) $$\pi_{s}=\frac{2|s^{\mathrm{rec}}\cap s^{*}|}{|s^{\mathrm{rec}}|+|s^{*}|}$$
  where $|s^{\mathrm{rec}}|$ and $|s^{*}|$ are the cardinality number of the observed species from inversion and data, respectively and $|s^{\mathrm{rec}}\cap s^{*}|$ is the cardinality number of the intersection between inverted species $s^{\mathrm{rec}}$ and species from data $s^{*}$.
- reconstruction Dice score for tumor core (Op∪On) denoted as tc
(32) $$\pi_{\mathrm{tc}}=\frac{2|{\mathrm{tc}}^{\mathrm{rec}}\cap {\mathrm{tc}}^{*}|}{|{\mathrm{tc}}^{\mathrm{rec}}|+|{\mathrm{tc}}^{*}|}$$
  where $|{\mathrm{tc}}^{\mathrm{rec}}|$ and $|{\mathrm{tc}}^{*}|$ are the cardinality number of tumor core from inversion and data, respectively and $|{\mathrm{tc}}^{\mathrm{rec}}\cap {\mathrm{tc}}^{*}|$ is the cardinality number of the intersection between inverted tumor core ${\mathrm{tc}}^{\mathrm{rec}}$ and tumor core from data ${\mathrm{tc}}^{*}$. Note that we denote the Dice score from single‐species model with $\pi_{\mathrm{tc}}^{\mathrm{ss}}$ and Dice score from multi‐species model with $\pi_{\mathrm{tc}}^{\mathrm{ms}}$.

### 3.3 1D Test‐Case

In this section, first, we discuss the ill‐posedness of the problem in 1D and then, we present the inversion results. We present the analysis of the 1D inversion assuming that the IC is known and we only need to invert for the growth coefficients.

#### 3.3.1 III‐Posedness Analysis

To address the ill‐posedness of the problem, we evaluate the Hessian for 3500 samples of q uniformly derived from coefficients range and perform an eigenvalue decomposition. The eigenvalue spectrum, displayed in Figure 2a, exhibits an exponential decrease, indicating that the problem is highly ill‐posed. The wide range of eigenvalues suggests that different combinations of coefficients contribute to the problem's ill‐posedness with varying degrees of difficulty to reconstruct the coefficients.

Following Algorithm 1, we further analyze the ill‐posed directions by performing singular value decomposition (SVD) on weighted eigenvectors. The result of the SVD is displayed in Figure 2b through the plot of the singular values. The smaller singular value suggests higher ill‐posedness of the corresponding coefficient combinations. In Figure 2c, we have illustrated the last four directions of the computed $U_{R}$, which correspond to the 4 smallest singular values from Figure 2b. It is crucial to consider the weight for penalizing the $\gamma_{0}$ parameter in the first direction. This penalization corresponds to the necrotic species and accounts for the fact that if a location is assigned to the necrotic region in both the data and observation, increasing the $\gamma_{0}$ coefficient will not reduce the error as the objective function is insensitive to it. Furthermore, we have observed through our simulations that high values of $\delta_{c}$ and ρ in the second and third directions contribute to the ill‐posedness.

#### 3.3.2 Inversion Analysis in 1D With Noise

In this section, we present results from inverting 1D synthetic cases, where exact IC is known and focus is on finding growth coefficients q. To test inversion stability, we generate species concentrations ($s^{*}$) using ground truth growth coefficients $q^{*}$, and we generate the noisy species ${\hat{s}}^{*}=s^{*}\left(1+\text{noise}\right)$ where *noise* has a zero‐mean Gaussian distribution with variance σ ($N\left(0,\sigma \right)$). We then apply the observation operators to generate the noisy observed data. We vary σ to generate different average noise levels among all species p,n and i. This noisy data is then input for the inversion algorithm and tested with and without regularization to evaluate stability and performance of reconstruction.

**FIGURE 3:**
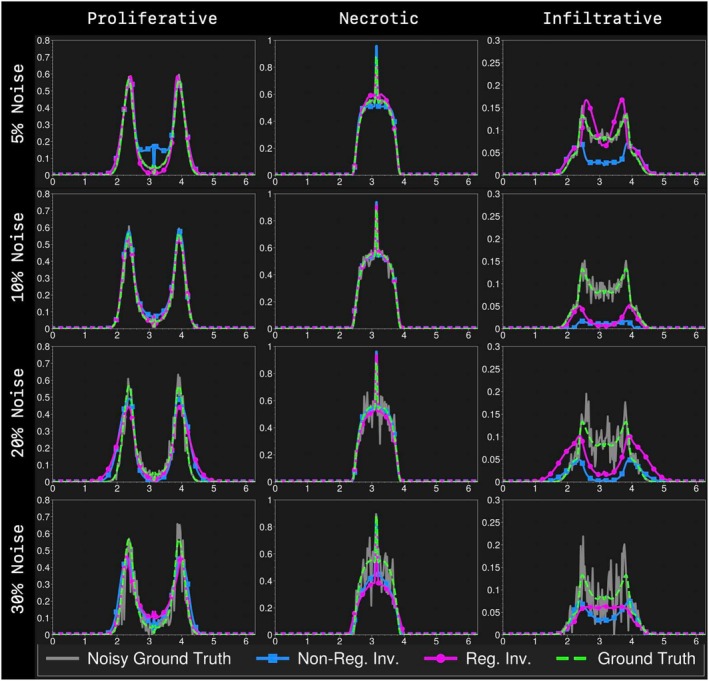
1D inversion results of synthetic data with additive Gaussian noise (5%,10%,20%,30%). Here we show the reconstructed species concentration at T=1. First to fourth rows show relative noise levels. Plots in first to third columns show proliferative, necrotic, and infiltrative species, respectively. The green, gray, magenta, and blue lines represent the underlying ground truth species (before observation denoted as “Ground Truth”), ground truth species with noise (“Noisy Ground Truth”), reconstructed species without regularization (“Non‐Reg. Inv.”), and reconstructed species with regularization (“Reg. Inv.”), respectively. Regularized inversion is observed to better match the underlying species even when the data is perturbed by noise, particularly in the case of infiltrative cells where deviations are greatly reduced with regularization.

In Figure 3, we show reconstructed species concentrations (proliferative p, infiltrative i, necrotic n) for synthetic cases with different levels of independent Gaussian noise added to each signal. The results with and without regularization are displayed. For a quantitative comparison, see Table 3 where regularization generally improves reconstruction performance compared to non‐regularized results. In Table 3, we also observe that regularization avoids parameter inversion problems, as seen in the case with 10% noise where some parameters diverge by comparing the average relative errors for parameters reconstruction. Table A1 reports inverted coefficients for each parameter with and without regularization. Overall, the inversion scheme with regularization is more stable in terms of species reconstruction and parameter inversion.

**TABLE 3:** Evaluation of 1D inversion scheme for the model parameters with synthetic data assuming known initial condition for the proliferative tumor cells.

Case	Noise (%)	$\pi_{n}$	$\pi_{p}$	$\pi_{l}$	$\mu_{n,L_{2}}$	$\mu_{p,L_{2}}$	$\mu_{i,L_{2}}$	$e_{q}$
Non‐Reg	5	$1.65\times 10^{-2}$	$9.36\times 10^{-1}$	$4.07\times 10^{-2}$	$7.74\times 10^{-2}$	$2.43\times 10^{-1}$	$5.99\times 10^{-1}$	$6.64\times 10^{-1}$
Reg	5	$9.86\times 10^{-1}$	$9.36\times 10^{-1}$	$5.80\times 10^{-1}$	$8.01\times 10^{-2}$	$1.45\times 10^{-1}$	$3.69\times 10^{-1}$	$5.23\times 10^{-1}$
Non‐Reg	10	$1.77\times 10^{-2}$	$9.34\times 10^{-1}$	$9.89\times 10^{-2}$	$4.91\times 10^{-2}$	$1.11\times 10^{-1}$	$8.73\times 10^{-1}$	$1.36\times 10^{0}$
Reg	10	$9.83\times 10^{-1}$	$9.29\times 10^{-1}$	$4.26\times 10^{-1}$	$5.79\times 10^{-2}$	$8.12\times 10^{-2}$	$7.59\times 10^{-1}$	$6.86\times 10^{-1}$
Non‐Reg	20	$1.50\times 10^{-2}$	$8.92\times 10^{-1}$	$5.59\times 10^{-2}$	$6.04\times 10^{-2}$	$2.89\times 10^{-1}$	$8.07\times 10^{-1}$	$6.76\times 10^{-1}$
Reg	20	$9.70\times 10^{-1}$	$8.99\times 10^{-1}$	$5.13\times 10^{-1}$	$9.86\times 10^{-2}$	$3.25\times 10^{-1}$	$6.87\times 10^{-1}$	$7.98\times 10^{-1}$
Non‐Reg	30	$6.85\times 10^{-2}$	$8.22\times 10^{-1}$	$4.43\times 10^{-2}$	$2.41\times 10^{-1}$	$2.64\times 10^{-1}$	$4.96\times 10^{-1}$	$4.65\times 10^{-1}$
Reg	30	$9.17\times 10^{-1}$	$8.32\times 10^{-1}$	$3.71\times 10^{-1}$	$3.17\times 10^{-1}$	$2.56\times 10^{-1}$	$4.14\times 10^{-1}$	$4.74\times 10^{-1}$

*Note:* Gaussian noise is added to species to generate noisy data. Dice scores of observed species s ($\pi_{s}$) and relative $L_{2}$ error of true species with inverted species s ($\mu_{s,L_{2}}$) are reported. Average of relative errors of growth coefficients q ($e_{p}$) is also reported. Regularization improves reconstruction error, especially for species and growth coefficients, though not for all noise levels. Nevertheless, regularization mainly stabilizes the inversion scheme, especially for proliferative cells, where the deviation of errors is more pronounced compared to the non‐regularized case. The Dice coefficients in the unregularized cases are higher because the unregularized model is overfitting to the noise in the data.

Further, we examine the influence of varying coefficient combinations on the performance of the inversion process. To achieve this objective, we conduct experiments on 150 synthetic data sets contaminated with 20% Gaussian noise and the results are presented in Table A2. The reconstruction measures are calculated both for regularized and non‐regularized inversion cases. Our results indicate that the use of regularization leads to a noticeable reduction in the average relative error of the coefficients ($e_{q}$), with an improvement of approximately 30%. This improvement is particularly noticeable for the coefficients $\gamma_{0},\beta_{0},$ and $\alpha_{0}$.

### 3.4 3D Test‐Cases

In this section, we analyze the 3D inversion. Results for cases with known brain anatomy (Ω) and IC ($p_{0}$) is presented first, where our goal is to estimate growth coefficients q only. The next case involves inverting for both IC and growth coefficients. Finally, results for real clinical data from BraTS20 training dataset [25] are presented.

#### 3.4.1 Artificial Tumor With Known IC and Anatomy

**FIGURE 4:**
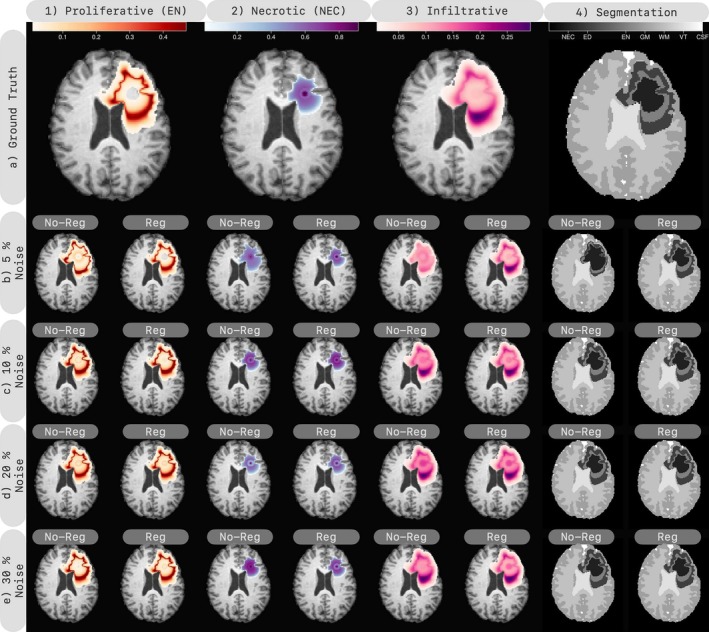
Evaluating 3D inversion using synthetic data with known IC and brain anatomy. We start by generating a tumor test‐case and showing tumor species including proliferative or enhancing (EN), necrotic (NEC) and edema (ED) and segmentation in the first row (a). We then add 5%, 10%, 20%, and 30% Gaussian noise to the species and apply an observation operator to get noisy data. We perform inversion with and without regularization on this data. The inversion results are displayed in rows 2–4, with the enhancing, necrotic, and infiltrative concentrations in columns 1–3, and the segmentation in the last column. The regularized inversion yields acceptable segmentation for all noise levels, whereas the non‐regularized case shows more variability for different noise levels.

In this section, we evaluate inversion for growth parameters only. Given a ground truth $q^{*}$, we solve the forward problem to generate data. We test different noise levels of 5%,10%,20%, and 30% and present the reconstruction with and without regularization in Figure 4. We report the results in Table 4 and individual model parameters and their relative errors in Table A3.

**TABLE 4:** Numerical evaluation of 3D inversion using synthetic data with known anatomy and IC.

Case	Noise (%)	$\pi_{n}$	$\pi_{p}$	$\pi_{l}$	$\mu_{n,L_{2}}$	$\mu_{p,L_{2}}$	$\mu_{i,L_{2}}$	$e_{q}$
Non‐Reg	5	$1.98\times 10^{-1}$	$5.72\times 10^{-1}$	$5.87\times 10^{-1}$	$9.95\times 10^{-1}$	$9.36\times 10^{-1}$	$4.21\times 10^{-1}$	$1.30\times 10^{0}$
Reg	5	$9.70\times 10^{-1}$	$9.81\times 10^{-1}$	$9.84\times 10^{-1}$	$4.41\times 10^{-2}$	$5.66\times 10^{-2}$	$2.91\times 10^{-2}$	$2.57\times 10^{-1}$
Non‐Reg	10	$9.23\times 10^{-1}$	$9.53\times 10^{-1}$	$9.52\times 10^{-1}$	$2.14\times 10^{-1}$	$2.51\times 10^{-1}$	$1.07\times 10^{-1}$	$7.58\times 10^{-1}$
Reg	10	$9.35\times 10^{-1}$	$9.58\times 10^{-1}$	$9.61\times 10^{-1}$	$2.07\times 10^{-1}$	$2.83\times 10^{-1}$	$8.90\times 10^{-2}$	$6.69\times 10^{-1}$
Non‐Reg	20	$9.57\times 10^{-1}$	$9.72\times 10^{-1}$	$9.73\times 10^{-1}$	$1.01\times 10^{-1}$	$1.44\times 10^{-1}$	$1.39\times 10^{-1}$	$9.42\times 10^{-1}$
Reg	20	$9.49\times 10^{-1}$	$9.67\times 10^{-1}$	$9.67\times 10^{-1}$	$1.18\times 10^{-1}$	$1.36\times 10^{-1}$	$1.21\times 10^{-1}$	$4.81\times 10^{-1}$
Non‐Reg	30	$9.24\times 10^{-1}$	$9.53\times 10^{-1}$	$9.44\times 10^{-1}$	$1.94\times 10^{-1}$	$3.40\times 10^{-1}$	$1.42\times 10^{-1}$	$3.76\times 10^{-1}$
Reg	30	$9.54\times 10^{-1}$	$9.71\times 10^{-1}$	$9.70\times 10^{-1}$	$1.06\times 10^{-1}$	$1.83\times 10^{-1}$	$1.13\times 10^{-1}$	$3.30\times 10^{-1}$

*Note:* We compare the Dice score of observed species $\pi_{s}$, the relative $L_{2}$ error of species $\mu_{s,L_{2}}$, and average relative error of coefficients $e_{q}$ for noise levels of 5%, 10%, 20%, and 30% with and without regularization. Regularization improves performance, especially for the 5% noise level in terms of coefficient error, although Dice scores do not vary drastically. The significance can be seen in the relative $L_{2}$ error of underlying species.

As seen in Table 4 and Figure 4, regularization noticeably improves the reconstruction error $e_{q}$ in 5% noise, while improving the reconstruction at higher noise levels. Although Dice scores and segmentation do not show substantial improvement, the underlying species reconstruction, not directly observed from data, is improved.

#### 3.4.2 Artificial Tumor With Unknown IC and Anatomy

In this section, we present the results for two‐stage inversion process for both IC ($p_{0}$) and growth coefficients (q). Our solution involves first the tumorous regions are filled with white matter and the healthy brain is estimated and we invert for the IC using a single‐species model, as detailed in [2, 3]. Once the IC is estimated, we estimate the growth coefficients.

**FIGURE 5:**
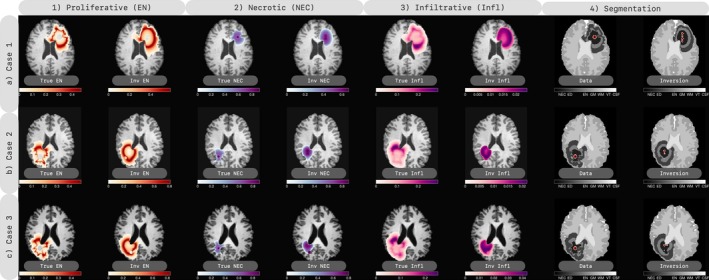
3D inversion result with synthetic test‐cases generated with an unknown IC and brain anatomy. Different normal brains with no mass deformation are used to generate the synthetic cases. In each row, we present a single test‐case, with tumor species (proliferative or enhancing (EN), necrotic (NEC), edema (ED) and infiltrative cells) from the synthetic cases and inversion displayed in the first, second, and third columns, respectively. The given data and segmentation computed by the inversion algorithm are depicted in the fourth column. Moreover, we depicted the projected ground truth IC and estimated IC in the segmentation for data and inversion, respectively, Although the IC is an estimate, the reconstruction from the inversion is good as evidenced by the qualitative observation. The challenge of reconstructing multi‐species arises due to the unknown initial brain anatomy and isotropic migration of tumor in all directions due to assigned white matter label.

Three cases are tested with generated data and different parameter combinations. The inversion results are depicted in Figure 5 and the corresponding numerical measures are reported in Table 5. The individual growth coefficients and their relative error are also included in Table A4.

**TABLE 5:** Quantitative evaluation of 3D inversion with unknown IC using synthetic data.

Case	$\pi_{\mathrm{tc}}^{\mathrm{ms}}$	$\pi_{\mathrm{tc}}^{\mathrm{ss}}$	$\pi_{p}$	$\pi_{n}$	$\pi_{l}$	$\mu_{n,L_{2}}$	$\mu_{p,L_{2}}$	$\mu_{i,L_{2}}$	$\mu_{0,L_{2}}$	$\mu_{0,\ell_{1}}$	$\mu_{0,\ell_{1}}^{x}$	$\mu_{0,\ell_{1}}^{y}$	$\mu_{0,\ell_{1}}^{z}$	$e_{q}$
Case 1	$8.43\times 10^{-1}$	$7.73\times 10^{-1}$	$6.46\times 10^{-1}$	$7.59\times 10^{-1}$	$7.85\times 10^{-1}$	$9.78\times 10^{-1}$	$5.08\times 10^{-1}$	$9.00\times 10^{-1}$	$1.18\times 10^{0}$	$1.60\times 10^{0}$	$1.14\times 10^{0}$	$1.12\times 10^{0}$	$1.05\times 10^{0}$	$4.28\times 10^{0}$
Case 2	$7.95\times 10^{-1}$	$7.35\times 10^{-1}$	$5.43\times 10^{-1}$	$7.74\times 10^{-1}$	$5.74\times 10^{-1}$	$1.28\times 10^{0}$	$8.78\times 10^{-1}$	$9.48\times 10^{-1}$	$1.10\times 10^{0}$	$1.33\times 10^{0}$	$1.06\times 10^{0}$	$6.82\times 10^{-1}$	$1.06\times 10^{0}$	$1.36\times 10^{0}$
Case 3	$8.19\times 10^{-1}$	$6.78\times 10^{-1}$	$6.13\times 10^{-1}$	$7.93\times 10^{-1}$	$7.20\times 10^{-1}$	$1.26\times 10^{0}$	$7.64\times 10^{-1}$	$8.94\times 10^{-1}$	$1.09\times 10^{0}$	$1.27\times 10^{0}$	$1.06\times 10^{0}$	$1.04\times 10^{0}$	$1.07\times 10^{0}$	$8.28\times 10^{-1}$

*Note:* The accuracy of species reconstruction is measured by the Dice score $\pi_{s}$, and the relative $L_{2}$ reconstruction of each species is reported as $\mu_{s,L_{2}}$. The relative $\ell_{1}$ and $L_{2}$ errors for the IC are indicated as $\mu_{0,L_{2}}$ and $\mu_{0,\ell_{1}}$, respectively, and the relative error in growth coefficients is denoted by $e_{q}$. We also report the projected error of IC estimation denoted as $\mu_{0,\ell_{1}}^{x}$, $\mu_{0,\ell_{1}}^{y}$ and $\mu_{0,\ell_{1}}^{z}$ in yz, xz, and xy planes, respectively. The results suggest that the Dice scores exhibit good accuracy in reconstructing the observed species. However, the combination of IC errors and coefficient errors substantially affects the relative error in the parameters, pointing to the need for further improvement.

As we can observe, we demonstrate that even though the IC estimation is derived from another model, it is still capable of reconstructing the observed tumor species from the data. Our test cases, as shown in Figure 5, indicate that the IC estimation provides a relatively good estimate for the initial condition. The estimation of the IC for a reaction–diffusion tumor growth model is exponentially ill‐posed, making it a challenging task, as noted in prior studies such as [2, 3].

As shown in Table 5, our solver not only achieves a reconstruction comparable to that of the single‐species model but also provides additional information on the underlying species. Our method enables the characterization of observed species and the estimation of non‐observable ones from the given data. As demonstrated in Section 3.4.1, our algorithm exhibits good convergence once a suitable estimate of IC is obtained. Notably, the performance of our method is influenced by the brain tumor anatomy, with the diffusion patterns substantially impacted by the tissue types in the test cases. Our model simplifies the problem by estimating the tumor growth model, given the challenge of accurately determining brain anatomy due to mass deformation. Furthermore, as illustrated in Figure 5, the diffusion of proliferative cells is primarily in all directions due to the allocation of white matter in the brain anatomy, while the data is influenced by the gray matter.

### 3.5 Inversion on Two Clinical Data

**FIGURE 6:**
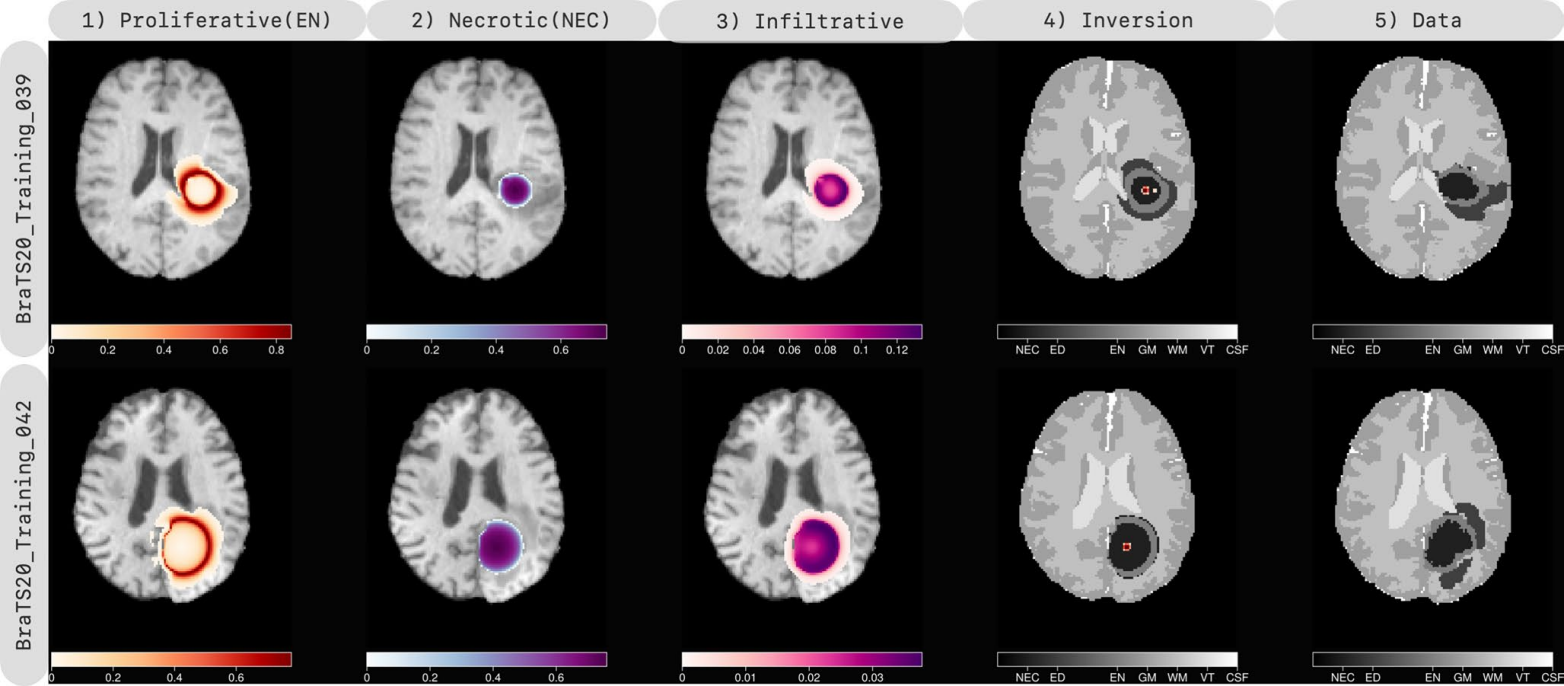
We conduct a multi‐species inversion of the BraTS20_Training clinical dataset, selecting two patients, using the MRI scan segmentation as input. Our results are presented in a tabular format, with each row showing the inversion and data for each patient. The first three columns depict the inverted species (proliferative or enhancing (EN), necrotic (NEC), and infiltrative cells), the fourth column shows the observed species from the inversion, and the last column displays the data. Our results demonstrate that, despite only having a single snapshot from each patient, we can qualitatively reconstruct the species distribution with reasonable accuracy.

In this section, we demonstrate the application of our inversion scheme to two patients from the BraTS20 dataset [25]. The procedure for reconstruction is outlined in Section 3.4.2 and involves first reconstructing IC of the tumor, followed by estimation of the growth parameters. The reconstructed species are visualized in Figure 6, with the projected IC estimate shown in the inverted segmentation. We report that the results can be found in Table 6 and Table A5. Note that the only information available from the patients is the segmentation of the observed species from MRI scans, and direct comparison of the underlying species concentrations is not possible.

**TABLE 6:** The results of the multi‐species model inversion using clinical data from the BraTS20 dataset: The performance of the model is measured by the Dice score, which serves as a quantitative measure of the accuracy of the reconstructed tumor species.

Patient's ID	$\pi_{\mathrm{tc}}^{\mathrm{ms}}$	$\pi_{\mathrm{tc}}^{\mathrm{ss}}$	$\pi_{n}$	$\pi_{p}$	$\pi_{l}$
BraTS20_Training_039	$8.23\times 10^{-1}$	$8.37\times 10^{-1}$	$6.94\times 10^{-1}$	$6.18\times 10^{-1}$	$4.24\times 10^{-1}$
BraTS20_Training_042	$9.01\times 10^{-1}$	$8.92\times 10^{-1}$	$7.25\times 10^{-1}$	$5.41\times 10^{-1}$	$0.00\times 10^{0}$

*Note:* The Dice score for the tumor core reconstruction by the multi‐species model is denoted as $\pi^{\mathrm{ms}}\mathrm{tc}$, while the Dice score for the tumor core reconstruction by the single‐species model is denoted as $\pi^{\mathrm{ss}}\mathrm{tc}$. Additionally, the Dice scores for the reconstruction of necrotic, proliferative, and edema regions are denoted as $\pi_{n}$, $\pi_{p}$, and $\pi_{l}$, respectively. As is evident from the results, our solver exhibits similar performance in reconstructing the tumor core compared to the single‐species model. Additionally, it provides the reconstruction of the individual species, offering added value in comparison to the single‐species approach.

Our solver achieves a reconstruction accuracy comparable to that of the single‐species model. Similar to the findings in Section 3.4.2. Furthermore, we are able to reconstruct species data and quantify infiltrative tumor cells. However, the reconstruction may be affected by IC estimation using the single‐species model, particularly for BraTS20_Training_039, as evidenced by the presence of a considerable amount of necrotic tissue in the initial condition location. Furthermore, the anisotropic progression of edema in BraTS20_Training_039 presents a challenging task for the solver to identify infiltrative cells. A similar trend can be observed for BraTS20_Training_042, where the edema does not uniformly diffuse. Our edema model is a simplified representation used in [1, 11]. Additionally, the results may be influenced by the species' volume in the data, as our unweighted objective function prioritizes reducing errors for the dominant species, that is, necrotic tissue in BraTS20_Training_042.

## 4 Conclusion

We presented an inverse solver for tumor growth model from one time snapshot and a regularization scheme to allow stable reconstruction of model parameters. The solver estimates the initial condition of the multi‐species model and growth coefficients. The input data is the MRI‐scan based tumor segmentation. Our solver uses just a single multiparametric scan, and we design a single‐snapshot inverse solver for the multi‐species model.

Our results demonstrate the efficacy of this regularization in improving the estimation of the growth coefficients in one‐dimensional and three‐dimensional settings, even in the presence of high relative noise. Moreover, we combine the regularization terms with our prior work on tumor initial condition estimation and apply the inversion algorithm to both synthetic and clinical cases, revealing the reconstruction's stability for the underlying species concentration. Our solver not only estimates the tumor core in a range comparable to that of the single‐species model, but also provides additional information on the reconstruction of other species. In this way, we can estimate the non‐observable species for the tumor growth model.

The solver is implemented using a scalable sampling optimization scheme, demonstrating a reasonable computational time. For initial condition estimation, Bayesian inference on the multispecies model with prior regularization can be used. However, such approaches may require many evaluations of the forward problem and its adjoint and are computationally prohibitive. Although the estimation of the initial condition could be improved (e.g., by using Bayesian inference with prior regularization, despite its high computational costs) to enhance the growth coefficients and reconstructions, our results provide valuable insights into the inverse problem for multi‐species tumor growth models.

In the future, we plan to conduct a comprehensive evaluation of the inversion scheme on a large number of clinical images to observe the correlation between the growth coefficients and clinical data outcomes, such as survival rate. As the model includes more detailed biophysical processes than the single‐species model, there are numerous opportunities for future extensions, including the inclusion of anisotropic diffusion and mass deformation for a more accurate modeling of GBM growth.

## Ethics Statement

The authors have nothing to report.

## Conflicts of Interest

The authors declare no conflicts of interest.

## Data Availability

The data that support the findings of this study are openly available in BraTS24 at https://www.synapse.org/Synapse:syn53708249/wiki/626323.

## Appendix A

**TABLE A1:** Inversion results of parameters using synthetically generated data in 1D.

Test‐case	Noise (%)	$e_{\kappa }\left(\frac{\kappa^{\mathrm{rec}}}{\kappa^{*}}\right)$	$e_{\rho }\left(\frac{\rho^{\mathrm{rec}}}{\rho^{*}}\right)$	$e_{\beta_{0}}\left(\frac{\beta_{0}^{\mathrm{rec}}}{\beta_{0}^{*}}\right)$	$e_{\alpha_{0}}\left(\frac{\alpha_{0}^{\mathrm{rec}}}{\alpha_{0}^{*}}\right)$	$e_{\gamma_{0}}\left(\frac{\gamma_{0}^{\mathrm{rec}}}{\gamma_{0}^{*}}\right)$	$e_{\delta_{c}}\left(\frac{\delta_{c}^{\mathrm{rec}}}{\delta_{c}^{*}}\right)$	$e_{\delta_{s}}\left(\frac{\delta_{s}^{\mathrm{rec}}}{\delta_{s}^{*}}\right)$	$e_{o_{h}}\left(\frac{{o_{h}}^{\mathrm{rec}}}{{o_{h}}^{*}}\right)$	$e_{o_{i}}\left(\frac{{o_{i}}^{\mathrm{rec}}}{{o_{i}}^{*}}\right)$	$e_{i_{e}}\left(\frac{i^{\mathrm{th},\mathrm{rec}}}{i^{\mathrm{th},*}}\right)$
Non‐Reg	5	$4.12\times 10^{-1}$ $\left(\frac{8.47\times 10^{-2}}{6.00\times 10^{-2}}\right)$	$1.53\times 10^{-1}$ $\left(\frac{1.61\times 10^{1}}{1.90\times 10^{1}}\right)$	$1.94\times 10^{-1}$ $\left(\frac{6.45}{8.00}\right)$	2.62 $\left(\frac{7.24}{2.00}\right)$	$2.92\times 10^{-2}$ $\left(\frac{1.03\times 10^{1}}{1.00\times 10^{1}}\right)$	$2.99\times 10^{-1}$ $\left(\frac{9.11}{1.30\times 10^{1}}\right)$	$5.42\times 10^{-1}$ $\left(\frac{1.37}{3.00}\right)$	$3.07\times 10^{-1}$ $\left(\frac{3.92\times 10^{-1}}{3.00\times 10^{-1}}\right)$	$2.16\times 10^{-1}$ $\left(\frac{3.92\times 10^{-1}}{5.00\times 10^{-1}}\right)$	$4.55\times 10^{-1}$ $\left(\frac{4.37\times 10^{-2}}{3.00\times 10^{-2}}\right)$
Reg	5	$4.28\times 10^{-1}$ $\left(\frac{8.57\times 10^{-2}}{6.00\times 10^{-2}}\right)$	$6.02\times 10^{-2}$ $\left(\frac{1.79\times 10^{1}}{1.90\times 10^{1}}\right)$	$2.02\times 10^{-1}$ $\left(\frac{6.38}{8.00}\right)$	$8.36\times 10^{-1}$ $\left(\frac{3.27\times 10^{-1}}{2.00}\right)$	2.00 $\left(\frac{3.00\times 10^{1}}{1.00\times 10^{1}}\right)$	$9.47\times 10^{-2}$ $\left(\frac{1.42\times 10^{1}}{1.30\times 10^{1}}\right)$	2.17 $\left(\frac{9.51}{3.00}\right)$	$2.53\times 10^{-1}$ $\left(\frac{3.76\times 10^{-1}}{3.00\times 10^{-1}}\right)$	$8.70\times 10^{-2}$ $\left(\frac{5.43\times 10^{-1}}{5.00\times 10^{-1}}\right)$	$5.15\times 10^{-1}$ $\left(\frac{4.54\times 10^{-2}}{3.00\times 10^{-2}}\right)$
Non‐Reg	10	$5.46\times 10^{-2}$ $\left(\frac{5.67\times 10^{-2}}{6.00\times 10^{-2}}\right)$	$2.56\times 10^{-1}$ $\left(\frac{2.39\times 10^{1}}{1.90\times 10^{1}}\right)$	1.37 $\left(\frac{1.89\times 10^{1}}{8.00}\right)$	$9.48\times 10^{-1}$ $\left(\frac{1.04\times 10^{-1}}{2.00}\right)$	$1.54\times 10^{-1}$ $\left(\frac{1.15\times 10^{1}}{1.00\times 10^{1}}\right)$	$7.25\times 10^{-1}$ $\left(\frac{3.57}{1.30\times 10^{1}}\right)$	$6.67\times 10^{-1}$ $\left(\frac{1.00}{3.00}\right)$	1.33 $\left(\frac{7.00\times 10^{-1}}{3.00\times 10^{-1}}\right)$	$9.66\times 10^{-1}$ $\left(\frac{9.83\times 10^{-1}}{5.00\times 10^{-1}}\right)$	$3.86\times 10^{-1}$ $\left(\frac{1.84\times 10^{-2}}{3.00\times 10^{-2}}\right)$
Reg	10	1.96 $\left(\frac{1.77\times 10^{-1}}{6.00\times 10^{-2}}\right)$	$1.13\times 10^{-1}$ $\left(\frac{2.11\times 10^{1}}{1.90\times 10^{1}}\right)$	2.72 $\left(\frac{2.98\times 10^{1}}{8.00}\right)$	$8.85\times 10^{-1}$ $\left(\frac{2.31\times 10^{-1}}{2.00}\right)$	1.81 $\left(\frac{2.81\times 10^{1}}{1.00\times 10^{1}}\right)$	$7.99\times 10^{-1}$ $\left(\frac{2.34\times 10^{1}}{1.30\times 10^{1}}\right)$	$6.66\times 10^{-1}$ $\left(\frac{1.00}{3.00}\right)$	$8.89\times 10^{-1}$ $\left(\frac{3.33\times 10^{-2}}{3.00\times 10^{-1}}\right)$	$7.91\times 10^{-2}$ $\left(\frac{4.60\times 10^{-1}}{5.00\times 10^{-1}}\right)$	3.63 $\left(\frac{1.39\times 10^{-1}}{3.00\times 10^{-2}}\right)$
Non‐Reg	20	2.02 $\left(\frac{1.81\times 10^{-1}}{6.00\times 10^{-2}}\right)$	$3.39\times 10^{-1}$ $\left(\frac{1.26\times 10^{1}}{1.90\times 10^{1}}\right)$	$4.94\times 10^{-1}$ $\left(\frac{4.05}{8.00}\right)$	$6.50\times 10^{-1}$ $\left(\frac{7.01\times 10^{-1}}{2.00}\right)$	$8.38\times 10^{-1}$ $\left(\frac{1.84\times 10^{1}}{1.00\times 10^{1}}\right)$	$4.18\times 10^{-1}$ $\left(\frac{7.57}{1.30\times 10^{1}}\right)$	$6.67\times 10^{-1}$ $\left(\frac{1.00}{3.00}\right)$	$7.13\times 10^{-1}$ $\left(\frac{5.14\times 10^{-1}}{3.00\times 10^{-1}}\right)$	$3.62\times 10^{-1}$ $\left(\frac{6.81\times 10^{-1}}{5.00\times 10^{-1}}\right)$	1.48 $\left(\frac{7.43\times 10^{-2}}{3.00\times 10^{-2}}\right)$
Reg	20	1.17 $\left(\frac{1.30\times 10^{-1}}{6.00\times 10^{-2}}\right)$	$1.03\times 10^{-1}$ $\left(\frac{1.70\times 10^{1}}{1.90\times 10^{1}}\right)$	$3.96\times 10^{-2}$ $\left(\frac{7.68}{8.00}\right)$	$9.50\times 10^{-1}$ $\left(\frac{1.00\times 10^{-1}}{2.00}\right)$	2.00 $\left(\frac{3.00\times 10^{1}}{1.00\times 10^{1}}\right)$	$4.57\times 10^{-1}$ $\left(\frac{7.06}{1.30\times 10^{1}}\right)$	$6.67\times 10^{-1}$ $\left(\frac{1.00}{3.00}\right)$	$5.84\times 10^{-1}$ $\left(\frac{4.75\times 10^{-1}}{3.00\times 10^{-1}}\right)$	$7.49\times 10^{-1}$ $\left(\frac{8.74\times 10^{-1}}{5.00\times 10^{-1}}\right)$	$3.26\times 10^{-2}$ $\left(\frac{3.10\times 10^{-2}}{3.00\times 10^{-2}}\right)$
Non‐Reg	30	$2.58\times 10^{-1}$ $\left(\frac{7.55\times 10^{-2}}{6.00\times 10^{-2}}\right)$	$7.49\times 10^{-2}$ $\left(\frac{1.76\times 10^{1}}{1.90\times 10^{1}}\right)$	$6.94\times 10^{-2}$ $\left(\frac{8.55}{8.00}\right)$	$5.99\times 10^{-1}$ $\left(\frac{8.02\times 10^{-1}}{2.00}\right)$	$6.32\times 10^{-1}$ $\left(\frac{3.68}{1.00\times 10^{1}}\right)$	$1.54\times 10^{-3}$ $\left(\frac{1.30\times 10^{1}}{1.30\times 10^{1}}\right)$	1.44 $\left(\frac{7.31}{3.00}\right)$	$9.98\times 10^{-1}$ $\left(\frac{5.99\times 10^{-1}}{3.00\times 10^{-1}}\right)$	$3.40\times 10^{-1}$ $\left(\frac{6.70\times 10^{-1}}{5.00\times 10^{-1}}\right)$	$3.29\times 10^{-1}$ $\left(\frac{2.01\times 10^{-2}}{3.00\times 10^{-2}}\right)$
Reg	30	$4.84\times 10^{-1}$ $\left(\frac{8.90\times 10^{-2}}{6.00\times 10^{-2}}\right)$	$2.42\times 10^{-2}$ $\left(\frac{1.85\times 10^{1}}{1.90\times 10^{1}}\right)$	$4.01\times 10^{-1}$ $\left(\frac{1.12\times 10^{1}}{8.00}\right)$	$7.94\times 10^{-1}$ $\left(\frac{4.13\times 10^{-1}}{2.00}\right)$	$4.19\times 10^{-1}$ $\left(\frac{5.81}{1.00\times 10^{1}}\right)$	$3.13\times 10^{-1}$ $\left(\frac{8.93}{1.30\times 10^{1}}\right)$	$6.64\times 10^{-1}$ $\left(\frac{1.01}{3.00}\right)$	$4.84\times 10^{-1}$ $\left(\frac{4.45\times 10^{-1}}{3.00\times 10^{-1}}\right)$	$8.02\times 10^{-1}$ $\left(\frac{9.01\times 10^{-1}}{5.00\times 10^{-1}}\right)$	$2.66\times 10^{-1}$ $\left(\frac{2.20\times 10^{-2}}{3.00\times 10^{-2}}\right)$

*Note:* In this table, we report the individual relative error for growth parameters for cases with noise levels. We report the relative error with regularization (Non‐Reg) and without regularization (Reg). We also report the true values and the inverted values in the parenthesis in front of each relative error.

**TABLE A2:** Inversion of synthetically generated data with 20% noise level for 150 parameters combinations with regularization.

Regularization	$e_{\kappa }$	$e_{\rho }$	$e_{\beta_{0}}$	$e_{\alpha_{0}}$	$e_{\gamma_{0}}$	$e_{\delta_{c}}$	$e_{\delta_{s}}$	$e_{o_{h}}$	$e_{o_{i}}$	$e_{i_{e}}$	$e_{q}$
Non‐Reg	1.42	$3.11\times 10^{-1}$	5.80	1.68	1.53	2.55	2.23	$9.24\times 10^{-1}$	$4.14\times 10^{-1}$	1.12	1.80
Reg	1.34	$2.90\times 10^{-1}$	3.78	1.16	1.09	2.86	2.11	1.00	$3.83\times 10^{-1}$	$9.33\times 10^{-1}$	1.50

*Note:* We report the average relative error for each individual parameter and also the average overall performance for all the parameters denoted as $e_{q}$.

**TABLE A3:** Inversion results for individual growth coefficients using synthetically generated data in 3D with known IC and brain anatomy.

Test‐case	Noise (%)	$e_{\kappa }\left(\frac{\kappa^{\mathrm{rec}}}{\kappa^{*}}\right)$	$e_{\rho }\left(\frac{\rho^{\mathrm{rec}}}{\rho^{*}}\right)$	$e_{\beta_{0}}\left(\frac{\beta_{0}^{\mathrm{rec}}}{\beta_{0}^{*}}\right)$	$e_{\alpha_{0}}\left(\frac{\alpha_{0}^{\mathrm{rec}}}{\alpha_{0}^{*}}\right)$	$e_{\gamma_{0}}\left(\frac{\gamma_{0}^{\mathrm{rec}}}{\gamma_{0}^{*}}\right)$	$e_{\delta_{c}}\left(\frac{\delta_{c}^{\mathrm{rec}}}{\delta_{c}^{*}}\right)$	$e_{\delta_{s}}\left(\frac{\delta_{s}^{\mathrm{rec}}}{\delta_{s}^{*}}\right)$	$e_{o_{h}}\left(\frac{{o_{h}}^{\mathrm{rec}}}{{o_{h}}^{*}}\right)$	$e_{o_{i}}\left(\frac{{o_{i}}^{\mathrm{rec}}}{{o_{i}}^{*}}\right)$	$e_{i_{e}}\left(\frac{i^{\mathrm{th},\mathrm{rec}}}{i^{\mathrm{th},*}}\right)$
Non‐Reg	5	$2.58\times 10^{-1}$ $\left(\frac{3.71\times 10^{-3}}{5.00\times 10^{-3}}\right)$	$2.28\times 10^{-1}$ $\left(\frac{1.84\times 10^{1}}{1.50\times 10^{1}}\right)$	2.51 $\left(\frac{7.01}{2.00}\right)$	1.99 $\left(\frac{5.99}{2.00}\right)$	$4.89\times 10^{-1}$ $\left(\frac{9.21}{1.80\times 10^{1}}\right)$	6.13 $\left(\frac{7.13}{1.00}\right)$	$6.44\times 10^{-3}$ $\left(\frac{1.79\times 10^{1}}{1.80\times 10^{1}}\right)$	1.11 $\left(\frac{4.22\times 10^{-1}}{2.00\times 10^{-1}}\right)$	$4.93\times 10^{-2}$ $\left(\frac{4.75\times 10^{-1}}{5.00\times 10^{-1}}\right)$	$1.83\times 10^{-1}$ $\left(\frac{2.37\times 10^{-2}}{2.00\times 10^{-2}}\right)$
Reg	5	$3.30\times 10^{-1}$ $\left(\frac{6.65\times 10^{-3}}{5.00\times 10^{-3}}\right)$	$2.47\times 10^{-3}$ $\left(\frac{1.50\times 10^{1}}{1.50\times 10^{1}}\right)$	$3.71\times 10^{-3}$ $\left(\frac{2.01}{2.00}\right)$	$7.39\times 10^{-2}$ $\left(\frac{2.15}{2.00}\right)$	$6.32\times 10^{-2}$ $\left(\frac{1.91\times 10^{1}}{1.80\times 10^{1}}\right)$	$7.53\times 10^{-1}$ $\left(\frac{1.75}{1.00}\right)$	$3.20\times 10^{-1}$ $\left(\frac{1.22\times 10^{1}}{1.80\times 10^{1}}\right)$	$7.26\times 10^{-1}$ $\left(\frac{3.45\times 10^{-1}}{2.00\times 10^{-1}}\right)$	$2.65\times 10^{-1}$ $\left(\frac{6.32\times 10^{-1}}{5.00\times 10^{-1}}\right)$	$2.87\times 10^{-2}$ $\left(\frac{2.06\times 10^{-2}}{2.00\times 10^{-2}}\right)$
Non‐Reg	10	$3.60\times 10^{-1}$ $\left(\frac{6.80\times 10^{-3}}{5.00\times 10^{-3}}\right)$	$5.47\times 10^{-2}$ $\left(\frac{1.58\times 10^{1}}{1.50\times 10^{1}}\right)$	$3.66\times 10^{-2}$ $\left(\frac{2.07}{2.00}\right)$	$1.56\times 10^{-1}$ $\left(\frac{2.31}{2.00}\right)$	$2.21\times 10^{-1}$ $\left(\frac{1.40\times 10^{1}}{1.80\times 10^{1}}\right)$	6.14 $\left(\frac{7.14}{1.00}\right)$	$1.10\times 10^{-1}$ $\left(\frac{2.00\times 10^{1}}{1.80\times 10^{1}}\right)$	$1.82\times 10^{-1}$ $\left(\frac{2.36\times 10^{-1}}{2.00\times 10^{-1}}\right)$	$2.97\times 10^{-1}$ $\left(\frac{3.51\times 10^{-1}}{5.00\times 10^{-1}}\right)$	$1.53\times 10^{-2}$ $\left(\frac{1.97\times 10^{-2}}{2.00\times 10^{-2}}\right)$
Reg	10	$4.01\times 10^{-1}$ $\left(\frac{7.01\times 10^{-3}}{5.00\times 10^{-3}}\right)$	$4.51\times 10^{-2}$ $\left(\frac{1.57\times 10^{1}}{1.50\times 10^{1}}\right)$	$2.72\times 10^{-2}$ $\left(\frac{2.05}{2.00}\right)$	$3.68\times 10^{-1}$ $\left(\frac{2.74}{2.00}\right)$	$3.86\times 10^{-2}$ $\left(\frac{1.73\times 10^{1}}{1.80\times 10^{1}}\right)$	3.72 $\left(\frac{4.72}{1.00}\right)$	$5.10\times 10^{-1}$ $\left(\frac{8.82}{1.80\times 10^{1}}\right)$	1.38 $\left(\frac{4.76\times 10^{-1}}{2.00\times 10^{-1}}\right)$	$1.92\times 10^{-1}$ $\left(\frac{5.96\times 10^{-1}}{5.00\times 10^{-1}}\right)$	$5.10\times 10^{-3}$ $\left(\frac{1.99\times 10^{-2}}{2.00\times 10^{-2}}\right)$
Non‐Reg	20	$4.76\times 10^{-1}$ $\left(\frac{7.38\times 10^{-3}}{5.00\times 10^{-3}}\right)$	$2.58\times 10^{-3}$ $\left(\frac{1.50\times 10^{1}}{1.50\times 10^{1}}\right)$	$2.81\times 10^{-2}$ $\left(\frac{1.94}{2.00}\right)$	$8.59\times 10^{-2}$ $\left(\frac{2.17}{2.00}\right)$	$3.59\times 10^{-1}$ $\left(\frac{1.15\times 10^{1}}{1.80\times 10^{1}}\right)$	6.95 $\left(\frac{7.95}{1.00}\right)$	$2.10\times 10^{-1}$ $\left(\frac{1.42\times 10^{1}}{1.80\times 10^{1}}\right)$	1.04 $\left(\frac{4.08\times 10^{-1}}{2.00\times 10^{-1}}\right)$	$2.10\times 10^{-1}$ $\left(\frac{6.05\times 10^{-1}}{5.00\times 10^{-1}}\right)$	$6.10\times 10^{-2}$ $\left(\frac{2.12\times 10^{-2}}{2.00\times 10^{-2}}\right)$
Reg	20	$1.44\times 10^{-1}$ $\left(\frac{5.72\times 10^{-3}}{5.00\times 10^{-3}}\right)$	$2.72\times 10^{-2}$ $\left(\frac{1.54\times 10^{1}}{1.50\times 10^{1}}\right)$	$1.93\times 10^{-2}$ $\left(\frac{1.96}{2.00}\right)$	$5.91\times 10^{-2}$ $\left(\frac{2.12}{2.00}\right)$	$3.34\times 10^{-1}$ $\left(\frac{1.20\times 10^{1}}{1.80\times 10^{1}}\right)$	3.60 $\left(\frac{4.60}{1.00}\right)$	$6.75\times 10^{-2}$ $\left(\frac{1.68\times 10^{1}}{1.80\times 10^{1}}\right)$	$4.94\times 10^{-1}$ $\left(\frac{2.99\times 10^{-1}}{2.00\times 10^{-1}}\right)$	$4.58\times 10^{-2}$ $\left(\frac{4.77\times 10^{-1}}{5.00\times 10^{-1}}\right)$	$1.93\times 10^{-2}$ $\left(\frac{2.04\times 10^{-2}}{2.00\times 10^{-2}}\right)$
Non‐Reg	30	1.09 $\left(\frac{1.05\times 10^{-2}}{5.00\times 10^{-3}}\right)$	$3.53\times 10^{-2}$ $\left(\frac{1.55\times 10^{1}}{1.50\times 10^{1}}\right)$	$3.58\times 10^{-2}$ $\left(\frac{1.93}{2.00}\right)$	1.13 $\left(\frac{4.25}{2.00}\right)$	$7.54\times 10^{-2}$ $\left(\frac{1.66\times 10^{1}}{1.80\times 10^{1}}\right)$	$6.23\times 10^{-1}$ $\left(\frac{1.62}{1.00}\right)$	$7.56\times 10^{-2}$ $\left(\frac{1.94\times 10^{1}}{1.80+10^{1}}\right)$	$4.67\times 10^{-2}$ $\left(\frac{1.91\times 10^{-1}}{2.00\times 10^{-1}}\right)$	$5.45\times 10^{-1}$ $\left(\frac{2.28\times 10^{-1}}{5.00\times 10^{-1}}\right)$	$1.00\times 10^{-1}$ $\left(\frac{2.20\times 10^{-2}}{2.00\times 10^{-2}}\right)$
Reg	30	$1.67\times 10^{-3}$ $\left(\frac{4.99\times 10^{-3}}{5.00\times 10^{-3}}\right)$	$4.12\times 10^{-2}$ $\left(\frac{1.56\times 10^{1}}{1.50\times 10^{1}}\right)$	$6.87\times 10^{-2}$ $\left(\frac{1.86}{2.00}\right)$	$1.23\times 10^{-1}$ $\left(\frac{1.75}{2.00}\right)$	$7.73\times 10^{-2}$ $\left(\frac{1.94\times 10^{1}}{1.80\times 10^{1}}\right)$	2.65 $\left(\frac{3.65}{1.00}\right)$	$1.30\times 10^{-2}$ $\left(\frac{1.82\times 10^{1}}{1.80\times 10^{1}}\right)$	$1.63\times 10^{-1}$ $\left(\frac{2.33\times 10^{-1}}{2.00\times 10^{-1}}\right)$	$9.69\times 10^{-2}$ $\left(\frac{4.52\times 10^{-1}}{5.00\times 10^{-1}}\right)$	$6.69\times 10^{-2}$ $\left(\frac{2.13\times 10^{-2}}{2.00\times 10^{-2}}\right)$

*Note:* We report the relative error for each growth coefficient, and we report the inverted parameters and the true growth coefficient.

**TABLE A4:** Inversion results of growth coefficients for synthetically generated data in 3D with unknown IC and brain anatomy.

Test‐case	$e_{\kappa }\left(\frac{\kappa^{\mathrm{rec}}}{\kappa^{*}}\right)$	$e_{\rho }\left(\frac{\rho^{\mathrm{rec}}}{\rho^{*}}\right)$	$e_{\beta_{0}}\left(\frac{\beta_{0}^{\mathrm{rec}}}{\beta_{0}^{*}}\right)$	$e_{\alpha_{0}}\left(\frac{\alpha_{0}^{\mathrm{rec}}}{\alpha_{0}^{*}}\right)$	$e_{\gamma_{0}}\left(\frac{\gamma_{0}^{\mathrm{rec}}}{\gamma_{0}^{*}}\right)$	$e_{\delta_{c}}\left(\frac{\delta_{c}^{\mathrm{rec}}}{\delta_{c}^{*}}\right)$	$e_{\delta_{s}}\left(\frac{\delta_{s}^{\mathrm{rec}}}{\delta_{s}^{*}}\right)$	$e_{o_{h}}\left(\frac{{o_{h}}^{\mathrm{rec}}}{{o_{h}}^{*}}\right)$	$e_{o_{i}}\left(\frac{{o_{i}}^{\mathrm{rec}}}{{o_{i}}^{*}}\right)$	$e_{i_{e}}\left(\frac{i^{\mathrm{th},\mathrm{rec}}}{i^{\mathrm{th},*}}\right)$
Case 1	$3.90\times 10^{1}$ $\left(\frac{2.00\times 10^{-1}}{5.00\times 10^{-3}}\right)$	$1.01\times 10^{-1}$ $\left(\frac{1.35\times 10^{1}}{1.50\times 10^{1}}\right)$	1.60 $\left(\frac{5.20}{2.00}\right)$	$6.46\times 10^{-1}$ $\left(\frac{7.09\times 10^{-1}}{2.00}\right)$	$9.59\times 10^{-2}$ $\left(\frac{1.63\times 10^{1}}{1.80\times 10^{1}}\right)$	$5.29\times 10^{-2}$ $\left(\frac{1.05}{1.00}\right)$	$1.07\times 10^{-1}$ $\left(\frac{1.99\times 10^{1}}{1.80\times 10^{1}}\right)$	$1.60\times 10^{-1}$ $\left(\frac{2.32\times 10^{-1}}{2.00\times 10^{-1}}\right)$	$3.53\times 10^{-1}$ $\left(\frac{3.24\times 10^{-1}}{5.00\times 10^{-1}}\right)$	$7.06\times 10^{-1}$ $\left(\frac{5.89\times 10^{-3}}{2.00\times 10^{-2}}\right)$
Case 2	5.41 $\left(\frac{3.21\times 10^{-2}}{5.00\times 10^{-3}}\right)$	$1.29\times 10^{-1}$ $\left(\frac{1.69\times 10^{1}}{1.50\times 10^{1}}\right)$	4.23 $\left(\frac{1.05\times 10^{1}}{2.00}\right)$	$6.37\times 10^{-1}$ $\left(\frac{7.27\times 10^{-1}}{2.00}\right)$	$3.34\times 10^{-2}$ $\left(\frac{1.74\times 10^{1}}{1.80\times 10^{1}}\right)$	$1.15\times 10^{-1}$ $\left(\frac{1.12}{1.00}\right)$	$6.38\times 10^{-1}$ $\left(\frac{6.52}{1.80\times 10^{1}}\right)$	1.44 $\left(\frac{4.88\times 10^{-1}}{2.00\times 10^{-1}}\right)$	$9.18\times 10^{-3}$ $\left(\frac{4.95\times 10^{-1}}{5.00\times 10^{-1}}\right)$	$9.50\times 10^{-1}$ $\left(\frac{1.00\times 10^{-3}}{2.00\times 10^{-2}}\right)$
Case 3	$8.65\times 10^{-1}$ $\left(\frac{9.32\times 10^{-3}}{5.00\times 10^{-3}}\right)$	$1.74\times 10^{-1}$ $\left(\frac{1.76\times 10^{1}}{1.50\times 10^{1}}\right)$	4.00 $\left(\frac{1.00\times 10^{1}}{2.00}\right)$	$4.49\times 10^{-1}$ $\left(\frac{1.10}{2.00}\right)$	$5.23\times 10^{-2}$ $\left(\frac{1.71\times 10^{1}}{1.80\times 10^{1}}\right)$	$3.59\times 10^{-1}$ $\left(\frac{1.36}{1.00}\right)$	$5.07\times 10^{-1}$ $\left(\frac{8.87}{1.80\times 10^{1}}\right)$	$7.25\times 10^{-1}$ $\left(\frac{3.45\times 10^{-1}}{2.00\times 10^{-1}}\right)$	$1.95\times 10^{-1}$ $\left(\frac{4.03\times 10^{-1}}{5.00\times 10^{-1}}\right)$	$9.50\times 10^{-1}$ $\left(\frac{1.00\times 10^{-3}}{2.00\times 10^{-2}}\right)$

*Note:* In this table, we report relative error for individual growth coefficients and also we report the inverted growth coefficients and the ground truth growth coefficients in the parenthesis in front of errors.

**TABLE A5:** Inversion results for growth coefficients using clinical data.

Patient‐ID	κ	ρ	$\beta_{0}$	$\alpha_{0}$	$\gamma_{0}$	$\delta_{c}$	$\delta_{s}$	$o_{h}$	$o_{i}$	$i_{e}$
BraTS20_Training_039	$1.21\times 10^{-3}$	$1.50\times 10^{1}$	$1.49\times 10^{1}$	9.98	$1.95\times 10^{1}$	1.42	8.24	$2.64\times 10^{-1}$	$2.65\times 10^{-1}$	$1.12\times 10^{-3}$
BraTS20_Training_042	$1.10\times 10^{-3}$	$1.88\times 10^{1}$	$1.50\times 10^{1}$	1.31	$1.47\times 10^{1}$	2.49	$1.41\times 10^{1}$	$3.15\times 10^{-1}$	$3.69\times 10^{-1}$	$2.15\times 10^{-2}$

*Note:* For each patient, we report the inverted growth coefficients using our inversion scheme.
